# The vaginal and fecal microbiomes are related to pregnancy status in beef heifers

**DOI:** 10.1186/s40104-019-0401-2

**Published:** 2019-12-13

**Authors:** Feilong Deng, Maryanna McClure, Rick Rorie, Xiaofan Wang, Jianmin Chai, Xiaoyuan Wei, Songjia Lai, Jiangchao Zhao

**Affiliations:** 1 Department of Animal Science, Division of Agriculture, University of Arkansas, Fayetteville, AR USA; 2 Farm Animal Genetic Resources Exploration and Innovation Key Laboratory of Sichuan Province, Sichuan Agricultural University, Chengdu, China; 3 Special Key Laboratory of Microbial Resources and Drug Development, Research Center for Medicine and Biology, Zunyi Medical University, Zunyi, China

**Keywords:** Beef cattle, Pregnancy, Random forest, Reproduction, Vaginal microbiome

## Abstract

**Background:**

The greatest impact on profitability of a commercial beef operation is reproduction. However, in beef heifers, little is known about the vaginal and fecal microbiota with respect to their relationship with fertility. To this end, we followed heifers through gestation to examine the dynamics of vaginal and fecal microbial composition throughout pregnancy.

**Results:**

Heifers were exposed to an estrus synchronization protocol, observed over a 12-day period, artificially inseminated 12 h to 18 h after observed estrus, and subsequently exposed to bulls for a 50-day breeding season. Vaginal samples were taken at pre-breeding (*n* = 72), during the first (*n* = 72), and second trimester (*n* = 72) for all individuals, and third trimester for individuals with confirmed pregnancies (*n* = 56). Fecal samples were taken at pre-breeding (*n* = 32) and during the first trimester (*n* = 32), including bred and open individuals. Next generation sequencing of the V4 region of the16S rRNA gene via the Illumina MiSeq platform was applied to all samples. Shannon indices and the number of observed bacterial features were the same in fecal samples. However, significant differences in vaginal microbiome diversity between gestation stages were observed. No differences in beta-diversity were detected in vaginal or fecal samples regarding pregnancy status, but such differences were seen with fecal microbiome over time. Random Forest was developed to identify predictors of pregnancy status in vaginal (e.g., *Histophilus*, Clostridiaceae, *Campylobacter*) and fecal (e.g., Bacteroidales, *Dorea*) samples.

**Conclusions:**

Our study shows that bovine vaginal and fecal microbiome could be used as biomarkers of bovine reproduction. Further experiments are needed to validate these biomarkers and to examine their roles in a female’s ability to establish pregnancy.

## Introduction

Reproduction has the greatest impact on profitability in commercial beef operations [1]. The losses in dairy and beef cattle due to reproductive failure results in a $1 billion annual loss in income for the cattle industry and makes reproductive failure six times more costly than the associated with respiratory disease [2]. Incorporating reproductive technologies, management strategies involving genetic selection and taking into account nutrition and seasonality can positively impact reproductive efficiency in a beef herd [3–5]. However, the less explored vaginal microbiota of the female bovine may also provide insights that help explain reproductive failure and success.

The extensively characterized human vaginal microbiome is divided into 5 community state types (CSTs) based on the dominating species of bacteria [6–8]. Four of these CSTs are dominated by the hydrogen peroxide and lactic acid producing genus of *Lactobacillus* species [9, 10]. In the fifth CST, the failure of *Lactobacillus* dominance can lead to the overgrowth of pathogenic bacteria resulting in bacterial vaginosis (BV) which is associated with adverse pregnancy outcomes [9, 11–13]. In non-human primates, the vaginal microbiome presents an increase in both richness and diversity [14]. The ewe vaginal microbiome is dominated by species of *Aggregatibacter*, *Streptobacillus*, *Cronobacter*, *Phocoenobacter* and *Psychrilyobacter* [15]. Unlike humans, the relative abundance of *Lactobacillus* species in the vaginal ecosystem is low at less than 3.5% and 0.53% for chimpanzees and ewes, respectively [14, 15].

In cattle, various studies report a variety of microbial compositions related to the vagina in female bovine. Laguardia-Nascimento et al. [16] characterized the vaginal microbiome of Nellore cattle using the next-generation sequencing approach and found the main bacterial phyla included Firmicutes (40–50%), Bacteroidetes (15–25%), and Proteobacteria (5–25%). Yeoman et al. [17] showed that the cattle vaginal microbiota harbor many rumen bacteria and methanogens, suggesting a possible role of the vagina in populating the rumen microbiome. In a recent study, Shpigel et al. [18] characterized the vaginal microbiome in the context of bovine necrotic vulvovaginitis (BNVV). They found increased abundance of Bacteroidetes and decreased community richness. They also identified indicator taxa for BNVV including *Parvimonas*, *Porphyromonas,* unclassified Veillonellaceae, *Mycoplasma* and Bacteroidetes [18]. Dominance by *Aggregatibacter*, *Streptobacillus, Phocoenobacter*, *Sediminicola* and *Sporobacter* species are reported in a study by Swartz et al. [15]. Members of Firmicutes, Bacteroidetes, *Ruminococcus*, *Dialister, Aeribacillus*, and *Porphyromonas* were dominant colonizers in a study reported by Gonzalez and colleagues [19]. Differences in relative abundance of certain genera in the vaginal microbiota in female bovine have been linked to reproductive disorders. Increased relative abundance in members affiliated with Bacteroides and Enterobacteriaceae (35.83% and 18.62%, respectively) have been shown in females with reproductive disease compared to healthy individuals with relative abundance values of 28.3% and 17.8%, respectively [20]. *Histophilus* has also been isolated from bovine vaginal communities diagnosed with a reproductive disorder, and not from those possessing a healthy reproductive function [20].

The interrelationship between hosts and their microbes is important in female fertility. In humans and non-human species alike, the suppression and over colonization of certain bacterial species in a niche results in disease pathogenicity and emphasizes the importance of understanding the interaction between host environment and its inhabiting microbes microbes [20–23]. It has been reported that *Lactobacillus* dominance is crucial to vaginal health in humans, but not other species [14]. Studies have shown positive effects of using probiotics to shift microbial communities in gestating humans to inhibit the growth of microbes that modify the host inflammatory response and signal for pre-term birth [24]. When ingested, these live organisms can alter the vaginal and gut microbiomes to produce metabolites and products that promote favorable metabolic activity during late stages of gestation [25]. Understanding the role and function of certain species of bacteria play in terms of fertility and overall reproductive performance in female cattle could help increase the reproductive fitness of cow herds worldwide.

Many steps will be needed to draw a causal relationship between vaginal microbiome and reproductive traits. Some of these steps include a first discovery type study to show the correlation, the isolation and re-inoculation of certain bacteria of interests into the vagina or the reduction of these bacteria to see the changes in reproductive traits. Therefore, the purpose of this study is the first step towards this end, to characterize the vaginal microbiome of commercial beef heifers and to determine if the vaginal microbiome could be used to predict the ability of a heifer to establish a pregnancy. Furthermore, this study seeks to understand the dynamic communities of vaginal environments in the gestating heifer by following individuals throughout pregnancy. Due to the important role that gut microbiome plays [26], we also included fecal microbiome in this study.

## Materials and methods

### Ethics statement

All animal work was approved and all methods were performed in accordance with guidelines of the Institutional Animal Care and Use Committee of the University of Arkansas under protocol # 16024. The University of Arkansas Division of Agriculture’s Beef Research Unit near Fayetteville, AR, housed 72 crossbred beef heifers averaging 420.88 ± 17.42 d of age and 328.036 ± 25.45 kg at the initiation of this study.

### Breeding strategy

At the onset of the breeding season, a 25 mg PGF2α injection (Lutalyse®, Zoetis, Parsippany, NJ) was administered intramuscularly in the neck. A heat detection patch (Estrotect Heat Patches®, Melrose, MN) was placed on the rump of each female. Heifers were then allocated to 1 of 6, 1 ha grass pastures. Each day for the subsequent 7 d, all heifers were monitored for estrus activity at 8:30 a.m. and 4:30 p.m.. Within 12 h to 18 h of detected estrus, heifers were artificially inseminated [27].

Following day 7 of estrus detection, those individuals not showing signs of estrus like behavior were administered a second PGF2α injection. This group of heifers were monitored and artificially inseminated as described above for 5 additional days. The remaining heifers were then moved to 6, 2.4 ha fescue-bermuda grass mixed pastures and were rotated every 28 d. Seven days after transfer to the pastures, a fertile bull was introduced to each pasture to initiate a 50-day breeding season. The bulls were rotated among the pasture every 7 d. A breeding soundness examination was performed on each bull no greater than 30 d before introduction to the heifer herd and following the 50 d breeding season to confirm fertility. After 50 d of exposure all bulls passed breeding soundness examinations.

Ultrasound was performed twice: on 63 d to determine pregnancies resulting from artificial insemination and on 60 d after bull removal to determine pregnancies resulting from bulls, when it is easy to identify AI versus bull bred pregnancies due to difference in crown-rump length (Additional file 1: Figure S1).

### Sample collection

At the onset of breeding season, fecal samples were deposited in 50 mL conical tubes and immediately placed on ice. The vulva was wiped clean with a paper towel and vaginal swabs were collected by inserting a double guarded culture swab (Jorgensen Labs, Loveland, Colorado, USA) at a 45^o^ angle into the vagina and moving to the posterior cervix. At the posterior cervix, the swab and inner guard were maneuvered through the outer guard. The swab was then pushed out of the inner guard and rolled on the surface of the vaginal epithelium for approximately 15 s. The swab was retracted back into the inner guard. The inner guard (containing the swab with sample) was retracted into the outer guard and the double guarded swab was removed from the animal. The swab was cut from the handle, placed in a 2-mL snap-cap tube with 1 mL of AMIES transport buffer and placed on ice. All samples were stored at −80 °C. Fecal and vaginal samples were taken from all individuals, as described previously at a second time point during the first trimester of gestation. Vaginal swabs were also taken from all heifers during the second trimester of gestation and again for those with confirmed pregnancies during the third gestational trimester (Additional file 1: Figure S1).

Detailed health records were maintained for each heifer throughout the entirety of the trial to ensure the health status of each individual. Each female was vaccinated with an inactivated vaccine containing IBR (BHV), BVD, BRSV, PI3, Leptospirosis and Vibriosis and treated for external and internal parasites according to the University of Arkansas Division of Agriculture’s Beef Research Unit cattle management protocol. Upon completion of the trial, pregnant heifers were maintained as one group and open heifers were culled. The retained females grazed on fescue-bermuda grass pastures and were supplemented with adequate free choice mineral supplements during gestation. Within 24 h of birth, calf sex and birthweight were recorded [27].

### DNA extraction and next-generation sequencing

Approximately 0.1 g of thawed feces was used for DNA extraction using the QIAamp PowerFecal DNA Kit (QIAGEN Inc., Germantown, MD, USA) according to the manufacturer’s protocol. DNA was extracted from the vaginal swabs using the QIAAmp BiOStic Bacteremia DNA Kit (QIAGEN Inc., Germantown, MD, USA) according to the manufacturer’s protocol. Nanodrop One C (Fisher Scientific, Hanover Park, IL, USA) was used to measure the DNA concentration and purity.

For library preparation, 10 ng of DNA were used for PCR amplification targeting the V4 region of the 16S rRNA gene. PCR was used to amplify each sample using dual index primers according to [28]. Amplicons were normalized using a SequalPrepTM Normalization Kit (Life Technologies, Grand Island, NY, USA) according to the manufacturer’s protocol. To generate the pooled library, 5 μL aliquots from each normalized sample (vaginal, *n* = 272; fecal, *n* = 64) were combined. The exact size of the library product and the concentration was measured with a KAPA Library Quantification Kit (Kapa Biosystems, Woburn, MA, USA) through quantitative PCR (Eppendorf, Westbury, NY, USA) assay and an Agilent 2100 Bioanalyzer System (Agilent, Santa Clara, CA, USA). The library was diluted based on the results from the qPCR and the bioanalyzer [27].

The 20 nmol/L pooled library, containing 336 individual samples, and a PhiX control v3 (20 nmol/L) (Illumina) were mixed with 0.2 N NaOH and HT1 buffer (Illumina). PhiX control v3 (5%, *v/v*) (Illumina) was added to the mix and 600 μL were loaded into a MiSeq® v2 (500 cycle) reagent cartridge for sequencing. The sequencing procedure was monitored periodically throughout the assay using the Illumina BaseSpace® website.

### Sequence analysis

The paired sequencing read files (R1 and R2) (approximately 250 base pairs in length) were downloaded to a local computer from the Illumina BaseSpace® website and the data was processed using the Deblur program integrated in the QIIME2 pipeline [29, 30]. Deblur obtains single-nucleotide resolution called amplicon sequence variants (ASVs), exact sequence variants (ESVs) or sub-operational taxonomic units (sub-OTUs) with statistical methods based on upper-bound error profiles within samples. Compared to other pipelines such as DADA2, UNOISE3 and open-reference OTU clustering at 97% similarity methods, Deblur tended to call the least amount of ASVs/OTUs [31], and is still robust for ecological assessment of microbiota [32]. We have similarly observed these patterns with our mock community (ZymoBIOMICs Microbial Community Standard, Zymo, Irvine, CA, USA) that contains eight bacterial taxa (Additional file 9: Table S1). The Deblur processed sequences were assigned to bacterial features, where features were different from each other at the single-nucleotide level. The features generated by Deblur could be compared between different studies. These features are synonymous to ASVs, ESVs and sub-OTUs. The Uchime algorithm was used to remove chimeric sequences [33]. Sequences were considered to be high quality if they have more than 90% of the bases with Phred score greater than 30 and passed the error reducing, chimera detection and removal steps. These features were classified using the naive Bayes method [34] and Greengenes (13_8 clustered at 99% similarity) database was used for the training of 16S Classifier. To reduce the effect of sequencing bias on the downstream alpha and beta diversity measures, the number of reads for fecal samples and vaginal swabs were rarefied to 3,000 and 1,000, respectively, which still resulted in saturated alpha diversity measures (Additional file 2: Figure S2).

### Ecological and statistical analyses

For all analyses, significance was determined as *P* < 0.05. Shannon Diversity index [35], and richness (number of observed OTUs) were calculated using QIIME2 to evaluate alpha diversity. The Kruskal-Wallis test was performed to explore differences in alpha diversities (Shannon Diversity index and richness) between heifers who established a pregnancy and those that did not, and over time for fecal and vaginal samples. Beta diversity was evaluated using Bray-Curtis [36] and Jaccard [37] distances, calculated in QIIME2, to explore the dissimilarity between the communities’ structure and membership, respectively. Random Forest was used to rank microbial signatures that accurately differentiate groups of female cattle. This machine learning technique accounts for non-linear relationships and dependencies among all microbial features. The relative abundance of the top 1,500 features and alpha-diversity measures were included as inputs for the Random Forest model. Each input (feature) was given an importance score (MDA: mean decrease accuracy) based on the increase in error caused by removing that feature from the predictors. Random forest uses about two-third of the samples in the dataset as a training set by randomly sampling with replacement and validate the selected features using the remaining “out-of-bag” samples.

## Results

### Vaginal microbial diversity increased from the pre-breeding season to the second trimester

A total of 336 samples were collected from commercial beef heifers prior to breeding and during each trimester of gestation. Vaginal (*n* = 272) and fecal (*n* = 64) were utilized for DNA extraction and sequencing of the V4 region of the 16S rRNA gene. After removing low quality reads and chimeras using QIIME 2 (2018.8), 3,617,919 and 1,584,626 high quality reads remained for vaginal and fecal samples respectively. Vaginal samples averaged 13,862 reads per sample ranging from 1153 to 98,623 (Additional file 10: Table S2). Fecal samples averaged 26,410 reads per sample ranging from 3045 to 453,279 (Additional file 10: Table S2). These sequences were assigned to 9496 and 4696 features based on 100% similarity for vaginal and fecal samples, respectively. The sequence number was normalized to 1,000 for vaginal samples and 3,000 for fecal samples to standardize sampling for downstream alpha and beta diversity analyses.

**Fig. 1:**
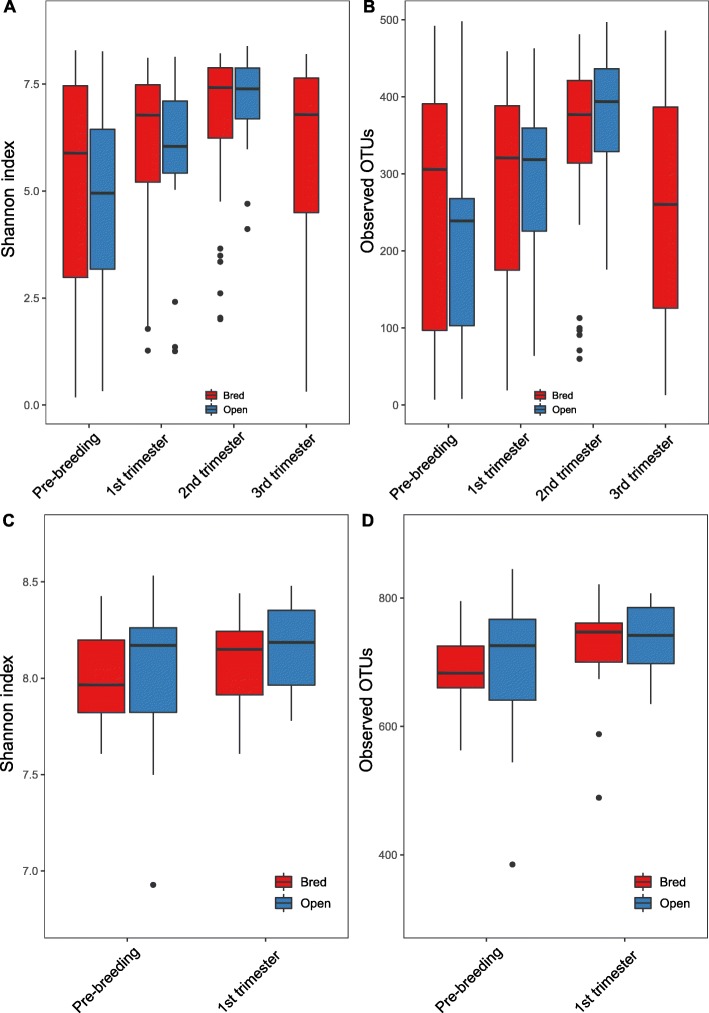
Vaginal and fecal microbial community alpha diversity measures between bred and open female cattle by stage. Diversity in the vaginal and fecal community was measured using Shannon index (**a**, **c**) and observed OTUs (**b**, **d**). The bottom and top of each box are the first and third quartiles, respectively, and the band inside the box is the median. Bred: cattle that were pregnant after the breeding season; Open: cattle that never established pregnancy. Vaginal and fecal swabs were collected at the sampling points (e.g. 1st trimester) from both pregnant and open cattle. The labels were defined based on the status of the pregnant cattle. Open cattle stayed open throughout the whole experiment

At the community level, although remarkable variation in the inter-animal dynamics of the alpha diversity was observed (Additional file 3: Figure S3), significant differences in the overall alpha diversity (Shannon index and the number of observed OTU’s) indices of the vaginal microbiome were observed over time (Fig. 1a, Kruskal-Wallis test, *P* = 6.475e-05, Fig. 1b, Kruskal-Wallis test, *P* = 3.149e-05). Microbial diversity (Shannon index) from both animals with and without established pregnancies increased from pre-breeding to the second trimester (*P* < 0.05, Table 1) and from the first trimester to the second trimester (*P* < 0.05, Table 1). The indices then decreased from the second to the third trimester (*P* < 0.05, Table 1). Regarding community richness (e.g., the number of observed OTUs), both open and bred individuals showed an increase in the number of observed OTUs from pre-breeding to the second trimester (*P* < 0.05, Table 1) and from the first trimester to the second (*P* < 0.05, Table 1). The number of observed OTUs decreased in bred females from the second trimester to the third (*P* < 0.05, Table 1). For fecal samples, we did not detect any significant differences in Shannon indices by pregnancy status or overall timeline (Fig. 1c, Kruskal-Wallis test, *P* = 0.53). Consistently, no significant differences in the total number of observed OTUs were observed in fecal samples (Fig. 1d, Kruskal-Wallis test, *P* = 0.24). *P* values for pairwise comparison of fecal samples are presented in Table 2.

**Table 1:** *P* values related to alpha diversity measures in vaginal samples based on pregnancy status^a^

	Comparison		Change in diversity	*P* value
Shannon index	1 Bred	1 Open		0.243
	2 Bred	2 Open		0.445
	3 Bred	3 Open		0.626
	1 Bred	2 Bred		0.119
	1 Bred	3 Bred	Increase	0.0005^*^
	1 Bred	4 Bred		0.146
	1 Open	2 Open		0.140
	1 Open	3 Open	Increase	0.001^*^
	2 Bred	3 Bred	Increase	0.018^*^
	2 Bred	4 Bred		0.935
	2 Open	3 Open	Increase	0.033^*^
	3 Bred	4 Bred	Decrease	0.047^*^
Observed OTUs	1 Bred	1 Open		0.158
	2 Bred	2 Open		0.962
	3 Bred	3 Open		0.437
	1 Bred	2 Bred		0.625
	1 Bred	3 Bred	Increase	0.002^*^
	1 Bred	4 Bred		0.876
	1 Open	2 Open		0.110
	1 Open	3 Open	Increase	0.002^*^
	2 Bred	3 Bred	Increase	0.003^*^
	2 Bred	4 Bred		0.419
	2 Open	3 Open	Increase	0.046
	3 Bred	4 Bred	Decrease	0.001^*^

^*^ Pair-wise comparisons between stage and pregnancy status were determined to be statistically significant at *P* < 0.05

^a^ Vaginal samples were obtained from 72 beef heifers. Individuals that established a pregnancy (*n* = 56) were samples before breeding (stage 1) and at 3 time points during gestation (stages 2, 3, and 4). Individuals that failed to establish a pregnancy (*n* = 16) were sampled before breeding (stage 1) and during the first and second trimesters of gestation (stages 2 and 3)

**Table 2:** *P* values related to alpha diversity measures in fecal samples based on pregnancy status^a^

	Comparison		*P* value
Shannon index	1 Bred	1 Open	0.4945
	2 Bred	2 Open	0.3519
	1 Bred	2 Bred	0.4773
	1 Open	2 Open	0.5249
Observed OTUs	1 Bred	1 Open	0.6073
	2 Bred	2 Open	0.8361
	1 Bred	2 Bred	0.085
	1 Open	2 Open	0.3084

^*^ Pair-wise comparisons between stage and pregnancy status were determined to be statistically significant at *P* < 0.05

^a^ Fecal samples were obtained from 32 beef heifers. Individuals that established a pregnancy (*n* = 16) and those that did not (*n* = 16) were sampled before breeding (stage 1) and during the first trimester (stage 2)

No significant differences in fecal or vaginal alpha diversity measures (Kruskal-Wallis, fecal: *P* = 0.59; vaginal: *P* = 0.155) between the open and the bred female cattle were observed at any time point.

### Vaginal and fecal microbiomes are indistinguishable based on the overall microbial membership and structure between open and bred cows

**Fig. 2:**
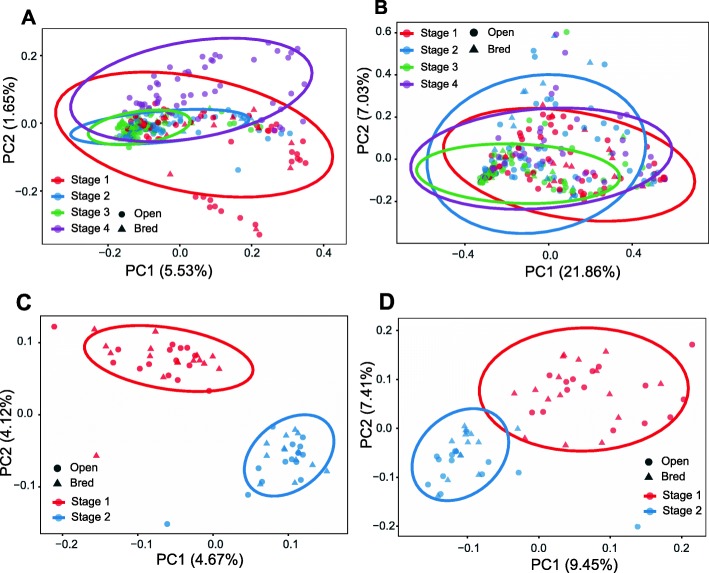
Beta diversity measures in vaginal (**a**, **b**) and fecal (**c**, **d**) samples across gestation stages and between open and bred cattle. **a**, **c** show the Principal Coordinate Analysis (PCoA) plot based on community membership as measured by the Jaccard distances. **b**, **d** show the PCoA based on community structure based on Bray-Curtis dissimilarity matrices. Triangles and circle represent bred and open females, respectively. Stages are indicated by color: red, blue, green and purple represent pre breeding, and gestational trimesters 1 through 3 respectively. These stages were defined based on the status of the pregnant cattle. Open cattle stayed open throughout the whole experiment. Samples were collected prospectively but pregnancy was defined retrospectively. The ellipses were calculated and drawn with 0.95 of confidence level. Bred: cattle that were pregnant after the breeding season; Open: cattle that never established pregnancy

We next examined dissimilarities in community membership and structure between pregnant and non-pregnant females overtime. The Jaccard dissimilarity matrix was used to evaluate bacterial community membership. To visualize the Jaccard distances, principal coordinate analysis (PCoA) was applied to the Jaccard dissimilarity matrix. Vaginal samples representative of all time points and each pregnancy status cluster together on principle coordinate axes 1 and 2 (PC1, PC2). No differences based on pregnancy status were detected (Fig. 2a, Analysis of Similarity, ANOSIM, stage 1: *P* = 0.542, R = − 0.018; stage 2: *P* = 0.805, R = − 0.075; stage 3: *P* = 0.856, R = − 0.099), but differences in community membership changed based on time (Fig. 2a, ANOSIM, R = 0.147, *P* ≤ 0.05). The Bray-Curtis index was used to estimate dissimilarities in both community membership and structure. PCoA plot based on Bray-Curtis distance shows no distinct clustering according to pregnancy status or time. No differences based on pregnancy status were seen (Fig. 2b, stage 1: *P* = 0.452, R = 0.008; stage 2: *P* = 0.673, R = − 0.029; stage 3: *P* = 0.825, R = − 0.063), but differences in community structure were observed based on time (Fig. 2b, ANOSIM, R = 0.138, *P* ≤ 0.05).

Interestingly, the PCoA plot based on Jaccard distance for fecal samples shows distinct clustering patterns over time (Fig. 2c, ANOSIM, R = 0.391, *P* < 0.001). No differences based on pregnancy status were observed (Fig. 2c, stage 1: *P* = 0.354, R = 0.011; stage 2: *P* = 0.418, R = 0.007). Consistently, significant differences in fecal community structure over time was demonstrated in the PCoA plot based on Bray-Curtis distances (Fig. 2d). No differences based on pregnancy status were seen (Fig. 2d, stage 1: *P* = 0.4, R = 0.006; stage 2: *P* = 0.789, R = − 0.029). Similar patterns were observed based on the PCoA plots based on unweighted UniFrac (Additional file 4: Figure S4).

### Remarkable inter-animal variation in community composition

**Fig. 3:**
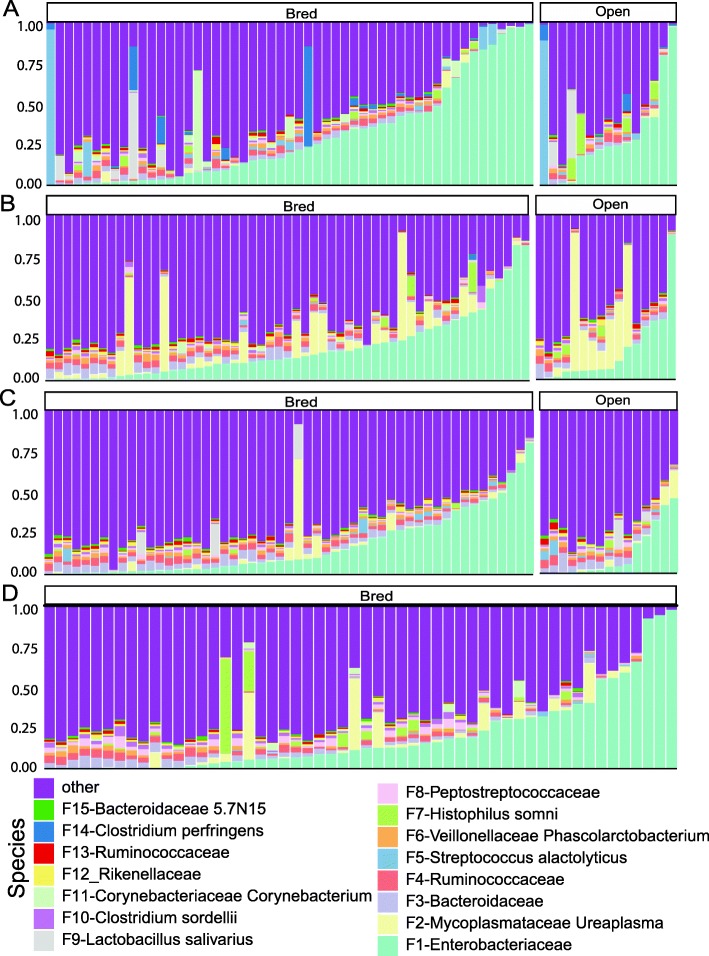
Relative abundance of bacterial features of different pregnancy status and stages in the vaginal microbiota of beef heifers. Multi-colored stacked bar graphs represent the relative abundance of the top 15 bacterial features. These features were classified against the Greengenes database and were shown at the deepest known classification. Each panel represents a stage (**a**: Pre-breeding, **b**: first trimester, **c**: second trimester, **d**: third trimester) and each bar represents a sample. These stages were defined based on the status of the pregnant cattle. Open cattle stayed open throughout the whole experiment

**Fig. 4:**
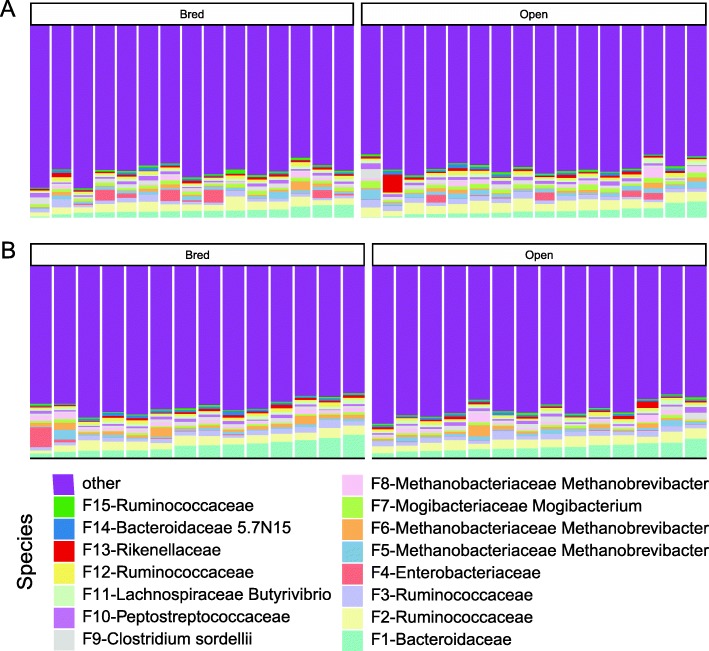
Relative abundance of bacterial features of different pregnancy status and stages in the fecal microbiota of beef heifers. Multi-colored stacked bar graphs represent the relative abundance of the top 15 bacterial features. Each panel represents a stage (**a**: Pre-breeding, **b**: first trimester) and each bar represents a sample. These stages were defined based on the status of the pregnant cattle. Open cattle stayed open throughout the whole experiment

The top 15 bacterial features of the bovine vaginal microbiome are shown in Fig. 3. The vaginal microbiome is dominated by an unclassified Enterobacteriaceae (21.05%), followed by *Ureaplasma* (4.37%) and an unclassified Bacteroidaceae (2.49%, Fig. 3). At the phylum level, Firmicutes was the most dominant taxon comprising 31.57%, followed by Proteobacteria (24.08%), Bacteroidetes (12.96%), and Tenericutes (4.95%, Additional file 5: Figure S5). These 4 phyla constituted 79.30% of the overall bacterial abundance (Additional file 5: Figure S5). In the fecal microbiome, the top 15 features includes several features associated with Ruminococcaceae and Bacteroidaceae (Fig. 4). At the phylum level, Firmicutes (45.93%), Bacteroidetes (18.83%), Euryarchaeota (6.14%) and Actinobacteria (2.57%) were 4 most abundant taxa, constituting 73.47% of the overall abundance in the fecal community (Additional file 6: Figure S6).

### Bacterial features are predictive of pregnancy status

**Fig. 5:**
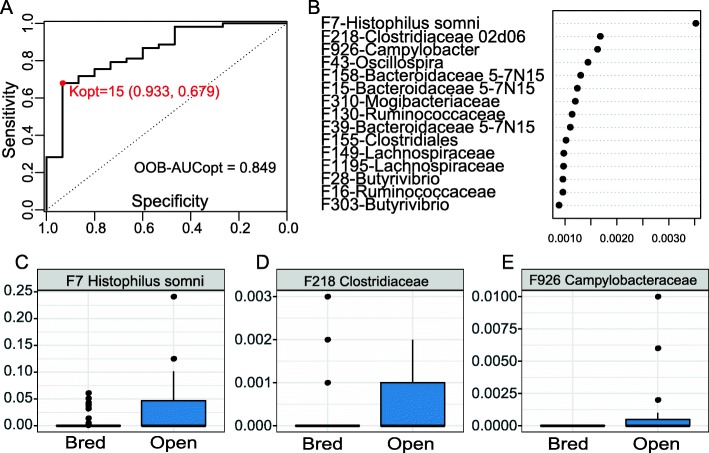
Predicting pregnancy outcome using random forest model with vaginal microbiota dataset of the pre-breeding stage. **a** ROC curve of the optimal random forest model. **b** Selected features in repeated cross-validation of the optimal random forest model. **c**-**e** Relative abundance of the top three features with the highest probability of selection

To assess if pre-breeding vaginal or fecal microbiome could be used to predict the success rate of pregnancy, we developed a Random Forest model to identify the bacterial features most predictive of pregnancy status. We determined the optimal model based on the maximum area under the curve (AUC) using the AUC-RF algorithm. For the vaginal microbiome, 15 features from the pre-breeding vaginal samples selected by random forest were able to predict if a cow could become pregnant with an AUC of 0.849 (sensitivity 0.933, specificity 0.679, Fig. 5a). The top 3 bacterial features including *Histophilus somni*, Clostridiaceae 02d06, and *Campylobacter* were more abundant in the open cows (Fig. 5b-e).

**Fig. 6:**
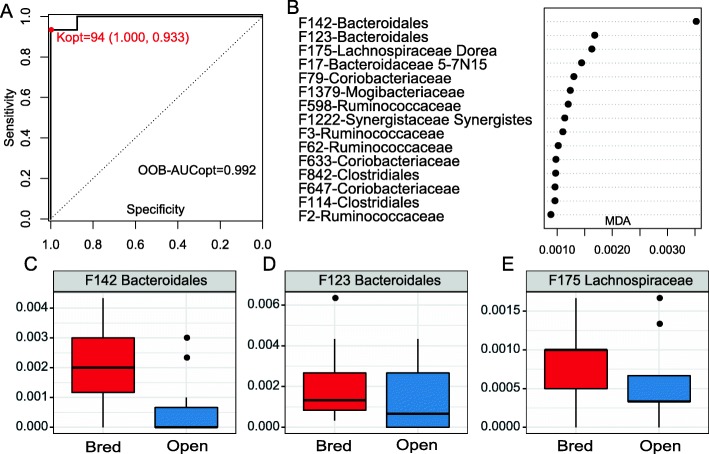
Predicting pregnancy outcome using Random forest model with fecal microbiota dataset of the pre-breeding stage. **a** ROC curve of the optimal random forest model with selected 94 features. **b** Top 15 features with the highest probability of selection in repeated cross-validation of the optimal random forest model. **c**-**e** Relative abundance of the three features with the highest probability of selection

Surprisingly, pre-breeding fecal microbiome also accurately predicted the capability of a cow to establish pregnancy after breeding with even higher accuracy (AUC = 0.992, sensitivity = 1.0, specificity = 0.933, Fig. 6a). Although 93 features were needed to obtain such a high accuracy, the top 15 features (Fig. 6b) alone yielded a very high AUC (0.929). The relative abundance and distribution of the top 3 features between the open and bred fecal samples are shown in Fig. 6c-e. All of the 3 features (2 associated with Bacteroidales and 1 with Lachnospiraceae) were more abundant in the feces of cows that established pregnancy after breeding.

Of note, most of these discriminant features persisted in subsequent time points (Additional file 7: Figure S7 and Additional file 8: Figure S8).

## Discussion

Relatively little is known about the microbes that inhabit the reproductive tract and their functions related to a female’s ability to reproduce. The bovine urogenital tract houses a variety of microbes composed of aerobic, facultative-anaerobic and anaerobic microorganisms [38]. There is much variation in this niche due to intrinsic and extrinsic factors, and little is known about the roles microbes play in reproduction [39].

No significant differences in bacterial community richness were observed in vaginal or fecal samples comparing bred and open females. In humans [40, 41], 4 of the 5 CST’s are dominated by different species of *Lactobacillus* leaving the fifth CST to be dominated by a mixture of strict and facultative anaerobes [7, 8]. Bacterial vaginosis (BV), which negatively impacts fertility, has a microbial composition similar to that of the fifth CST [42]. In a human study comparing the vaginal microbiota of subjects with and without clinically defined BV, those with BV presented an increase in taxonomic richness and diversity measured by the number observed OTU’s (*P* < 0.001) and the Shannon index being 1.4 to 4.1 times greater than those without BV [41]. The role *Lactobacillus* species play in the bovine vaginal ecosystem is yet to be determined, but it is possible that other species dominating the bovine vaginal niche have similar function.

Human vaginal microbiome studies have demonstrated the development of a more stable vaginal microbiota near the end of the gestation period. Aagaard et al. [40] reported decreased species richness and diversity that progressed with gestational age. A target set of *Lactobacillus* related OTUs are enriched in women with increased gestational age explaining changes in community membership and structure in late gestating humans [40].

Interestingly, although significant differences in bovine fecal microbial membership and structure over time were detected, no change in community membership or structure was observed in the vaginal niche of the female bovine throughout gestation, suggesting the bovine vaginal microbiome was stable and not affected by any of these factors. In addition, no changes or clusters were observed to differentiate pregnant from non-pregnant females in either vaginal or fecal samples at the community level.

In this study, the top 2 dominating features of the vaginal microbiota are affiliated with *Escherichia/Shigella*, and *Ureaplasma*. *Escherichia* has been documented as a contributing pathogen to metritis (uterine inflammation) due to its ability to establish residency in the reproductive tract from contamination by feces, ascend up the reproductive axis and maintain a presence in a contaminated uterus [43, 44]. In dairy cows, metritis is considered to be one of the most costly factors contributing to reproductive inefficiency due to increased days open, failure to conceive on the first service, increased number of inseminations, and failure to establish a pregnancy thus, establishing a link between *Escherichia* and reproductive failure [45]. *Ureaplasma* is a common isolate from cervicovaginal mucosal samples from beef females with healthy reproductive tracts [46]. However, *Ureaplasma* has been associated with cows suffering from granular vulvitis syndrome and mastitis, ciliostasis in cultured oviductal tissues and humans experiencing reproductive failure and infertility [47]. A previous study claims that *U. diversum* in combination with *Pasteurella* and/or *Manheimia* species causes lung lesions in calves resulting in pneumonia and consequent reoccurring morbidity [48]. This study agrees with the commonality of *Ureaplasma* isolation in vaginal samples, and since it’s presence is similar among bred and open females, this could explain a requirement for interaction with other pathogens to cause disease.

It is of particular interests to predict the likelihood of a heifer to establish a pregnancy based on her vaginal microbiome for improved reproduction strategies. Using Random Forest, we were able to identify 15 bacterial features that accurately (AUC = 0.849) differentiate heifers which established pregnancy from those that never did at the pre-breeding stage. Member of *Histophilus somni* was listed as the #1 predictor of pregnancy. Rodrigues and colleagues described the vaginal microbiome of female cattle with reproductive disorder. They found *Bacteroides*, *Enterobacteriaceae*, and *Histophilus* to be the top 3 dominant OTUs in unhealthy animals. Based on Random Forest predictors from this study, *Histophilus* can be used to predict the pregnancy status in vaginal samples before breeding. *Histophilus* species are gram-negative, non-spore-forming bacterium that can exist in both pathogenic and non-pathogenic forms [49]. Both forms of *H. somni* are isolated from the bovine mucous membranes of nasal passages, the prepuce and sheath of males and in the vagina of females [49]. Reproductive disease manifestation, most likely due to venereal spread, results in abortion, mastitis, and granular vulvovaginitis [49]. The increased abundance of *Histophilus* in females that do not establish a pregnancy in vaginal samples agrees with the presence of this OTU in animals with reproductive disorder and suggests the potential role that *Histophilus* plays in causing reproductive disorder. Clostridiaceae was observed in vaginal and uterine samples of animals and humans [6, 50, 51]. Certain species of Clostridiaceae were linked to bacterial vaginosis in humans [23, 51], but little is known about the function of Clostridiaceae in animal reproduction. *Campylobacter*, a clinical human and animal pathogen, can cause bovine venereal campylobacteriosis or vibriosis, which is the primary cause of abortion and infertility in cattle [52, 53]. In addition, *Campylobaacter* were also found as one of the most important vaginal bacteria causing abortion in sheep [54]. Feature 926, affiliated with *Campylobacter*, was listed as #3 predictor by Random Forest to predict pregnancy. This bacterium was only present in female cattle that did not establish pregnancy, which agrees with the reported infertility in heifers caused by *Campylobacter* infection.

Interestingly, bovine fecal microbiome predicted the establishment of pregnancy with even a higher accuracy than the vaginal microbiome (AUC = 0.929) with just 15 bacterial features. These features are associated with Bacteroidales, Ruminococcaceae, Coriobacteriaceae. Coriobacteriaceae has been isolated from the vagina of cattle with and without reproductive disorder, but it’s function is more accurately described in its symbiotic relationship with the gut of insects [20, 55]. This gram-positive, obligate anaerobe works to ferment glucose, and other compounds found in the foodstuffs of insects to produce lactic acid, ethanol, CO_2_ and H_2_ [55]. Three members of Coriobacteriaceae were listed as the top 15 predictors of pregnancy in the feces. The relative abundance of Coriobacteriaceae is smaller in cattle with pregnancy than in those that never establish pregnancy. The genus 5-7 N15 has been identified in nasopharyngeal [56], fecal [57, 58], milk [59] vaginal and uterine samples [50] of dairy cows or beef cattle. Mu et al. [57] showed the genus 5-7 N15 had a positive effect on milk production in Holstein dairy cows. In our study, members of 5-7 N15 were listed among the top 15 features that distinguish open versus bred cattle in both vaginal and fecal samples. More studies are needed to investigate the role of genus 5-7 N15 on cattle reproduction. Of note, despite its accurate prediction of the pregnancy status the causal relationship between gut microbiome and pregnancy has yet to be established. Colonization of the gut microbiome by contamination from the environment, or the feces, or possibly facilitated by intravaginal progesterone implant in the vagina has been reported [60]. A recent study showed the possible transmission of the gut microbiome to the uterus in cows by blood [61], which makes the connection between the gut microbiome and the reproductive tract. Nevertheless, given the many factors affecting pregnancy (e.g. cycling or not, timing of AI or breeding), the roles the gut microbiome plays in beef cattle production (if at all) need to be explored and validated by future experiments such as fecal microbiota transplant and/or inoculation of gut bacterial isolates into the GI or reproductive tract.

## Conclusion

In conclusion, the ability to use the vaginal microbiome in beef heifers to predict reproductive potential and gestational period is confirmed. Using Random Forest we identified specific bacterial strains that can predict pregnancy status, for both vaginal and fecal niches. Findings from this study advance the knowledge of the microbial communities residing in the vagina of beef heifers before breeding and throughout pregnancy.

## Supplementary information

**Additional file 1: Figure S1.** Flow chart of the experimental design and sample collection.

**Additional file 2: Figure S2.** Rarefaction curve. (A) Rarefaction plot of vaginal samples (one curve per group). (B) Rarefaction plot of fecal samples. 1, 2, 3 and 4 in figure legend represent the pregnancy stage of pre-breeding, first trimester, second trimester, and third trimester, respectively.

**Additional file 3: Figure S3.** Alpha diversity (Shannon index) change of vaginal microbiota for each sample. The bottom and top of each box are the first and third quartiles, respectively, and the band inside the box is the median. The connected point represent alpha diversity of animal vaginal samples at different stage.

**Additional file 4: Figure S4.** Principal Coordinates Analysis (PCoA) of unweighted UniFrac distances in vaginal (A) and fecal (B) samples across gestation stages and between open and bred cattle. Triangles and circle represent bred and open females, respectively. Stages are indicated by color: red, blue, green and purple represent pre breeding, and gestational trimesters 1 through 3 respectively. These stages were defined based on the status of the pregnant cattle. Open cattle stayed open throughout the whole experiment. Samples were collected prospectively but pregnancy was defined retrospectively. The ellipses were calculated and drawn with 0.95 of confidence level. Bred: cattle that were pregnant after the breeding season; Open: cattle that never established pregnancy.

**Additional file 5: Figure S5.** Relative abundance of vaginal microbiota at phylum level for each sample with different pregnancy status. Multi-colored stack bar graphs represent the relative abundance. Each chart represents a stage (A: Pre-breeding, B: first trimester, C: second trimester, D: third trimester).

**Additional file 6: Figure S6.** Relative abundance of fecal microbiota at phylum level for each sample with different pregnancy status. Multi-colored stack bar graphs represent the relative abundance. Each chart represents a stage (A: Pre-breeding, B: first trimester, C: second trimester, D: third trimester).

**Additional file 7: Figure S7.** Relative abundance of predictive bacterial features in vaginal samples across gestation stages and between open and bred cattle. 1, 2, 3 and 4 On the X-axis represent the pregnancy stage of pre-breeding, first trimester, second trimester, and third trimester, respectively.

**Additional file 8: Figure S8.** Relative abundance of predictive bacterial features in fecal samples at pre-breeding and first trimester and between open and bred cattle. 1, 2, 3 and 4 On the X-axis represent the pregnancy stage of pre-breeding, first trimester, second trimester, and third trimester, respectively.

**Additional file 9:** **Table S1.** Sequencing results of mock community.

**Additional file 10: Table S2.** Summary of sequencing and alpha diversity results for fecal and vaginal samples.

## Data Availability

The data generated were submitted to the National Center for Biotechnology Information (NCBI) Short Read Archive database (www.ncbi.nlm.nih.gov/sra) and are available with BioProject accession number PRJNA 497069.

## Abbreviations

AI: Artificial insemination
ANOSIM: Analysis of similarity
ASVs: Amplicon sequence variants
AUC: Area under the curve
BNVV: Bovine necrotic vulvovaginitis
BV: Bacterial vaginosis
CSTs: Community state types
ESVs: Exact sequence variants
MDA: Mean decrease accuracy
NCBI: National Center for Biotechnology Information
OTUs: Operational taxonomic units
PCoA: Principal coordinate analysis
sub-OTUs: Sub-operational taxonomic units
