# Supplementary figures and images for: The vaginal and fecal microbiomes are related to pregnancy status in beef heifers
Source: J Anim Sci Biotechnol. 2019 Dec 13;10:92. doi: 10.1186/s40104-019-0401-2 (PMC6909518; doi:10.1186/s40104-019-0401-2)

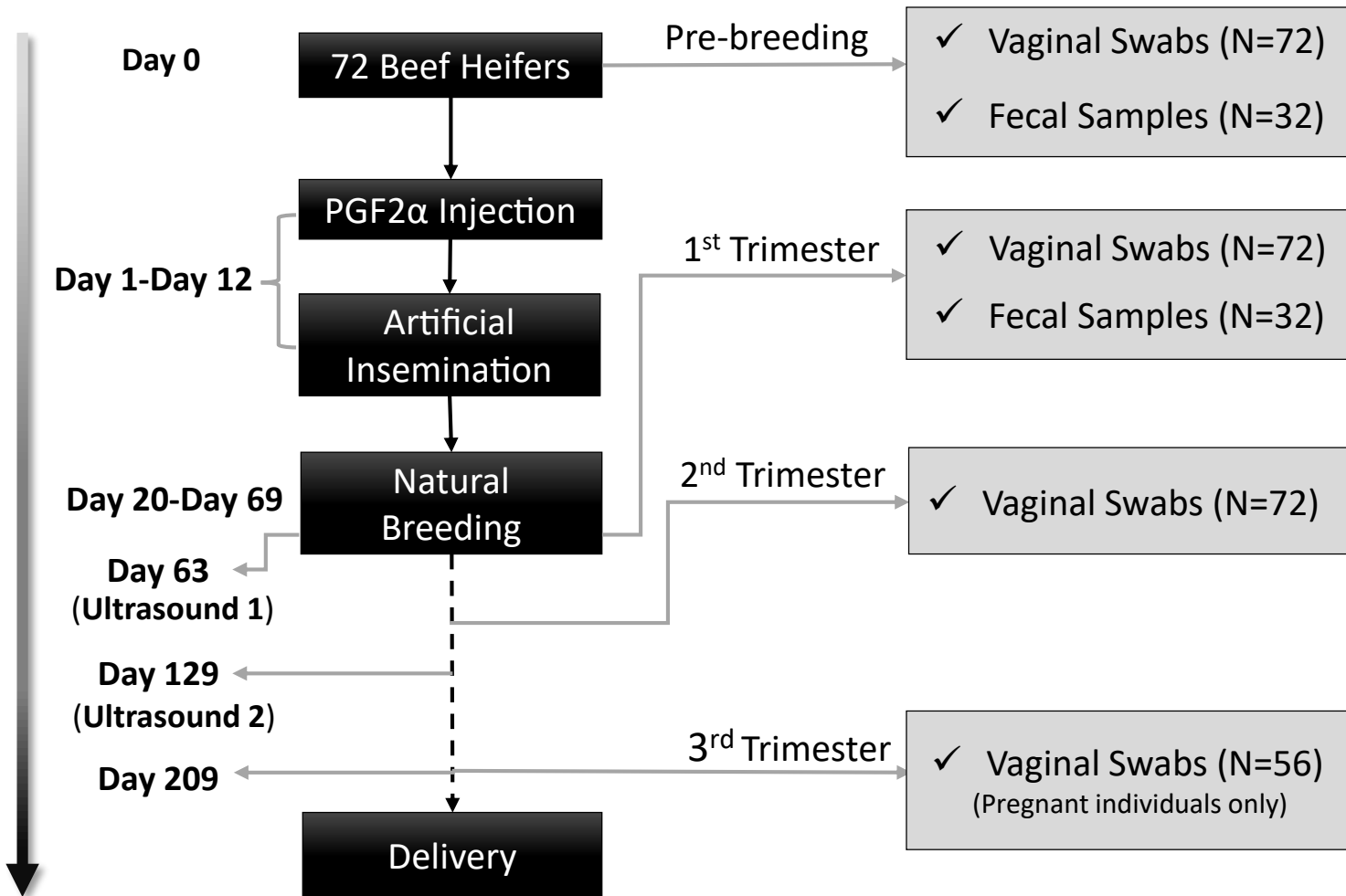

Supplement: Supplementary file 1 — Additional file 1: Figure S1. Flow chart of the experimental design and sample collection. [file 40104_2019_401_MOESM1_ESM.pdf]

A

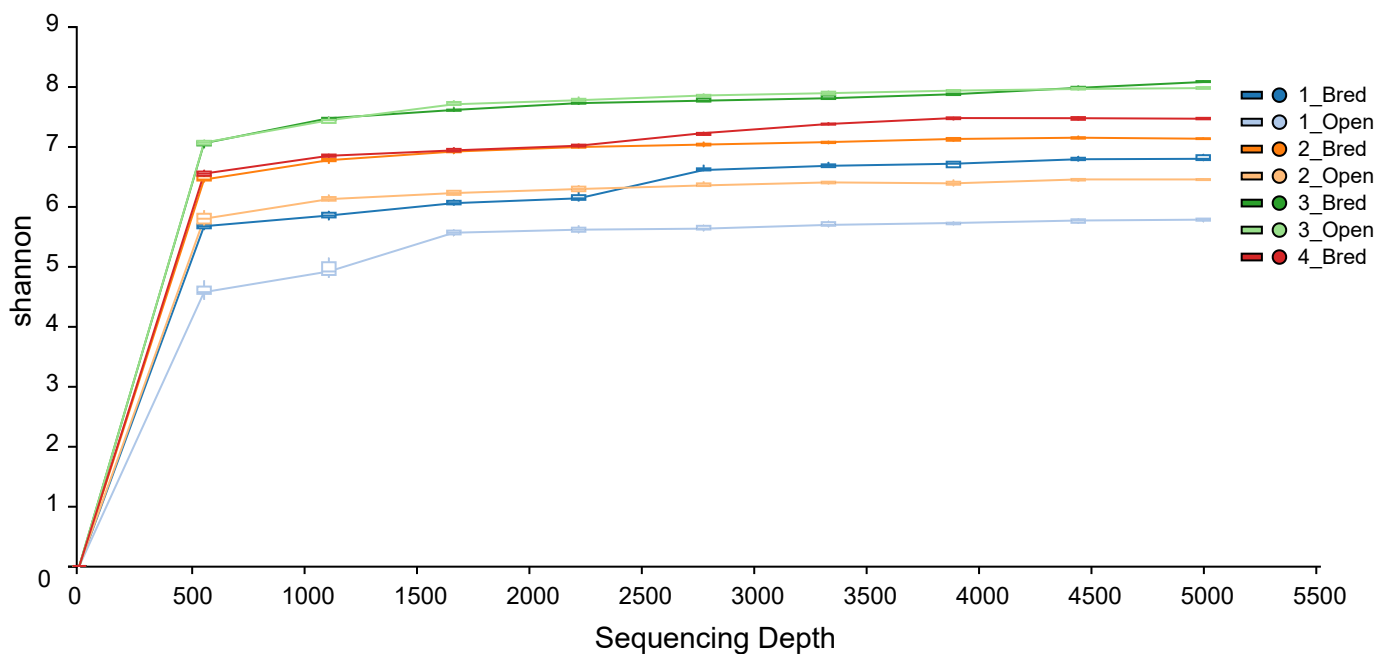

B

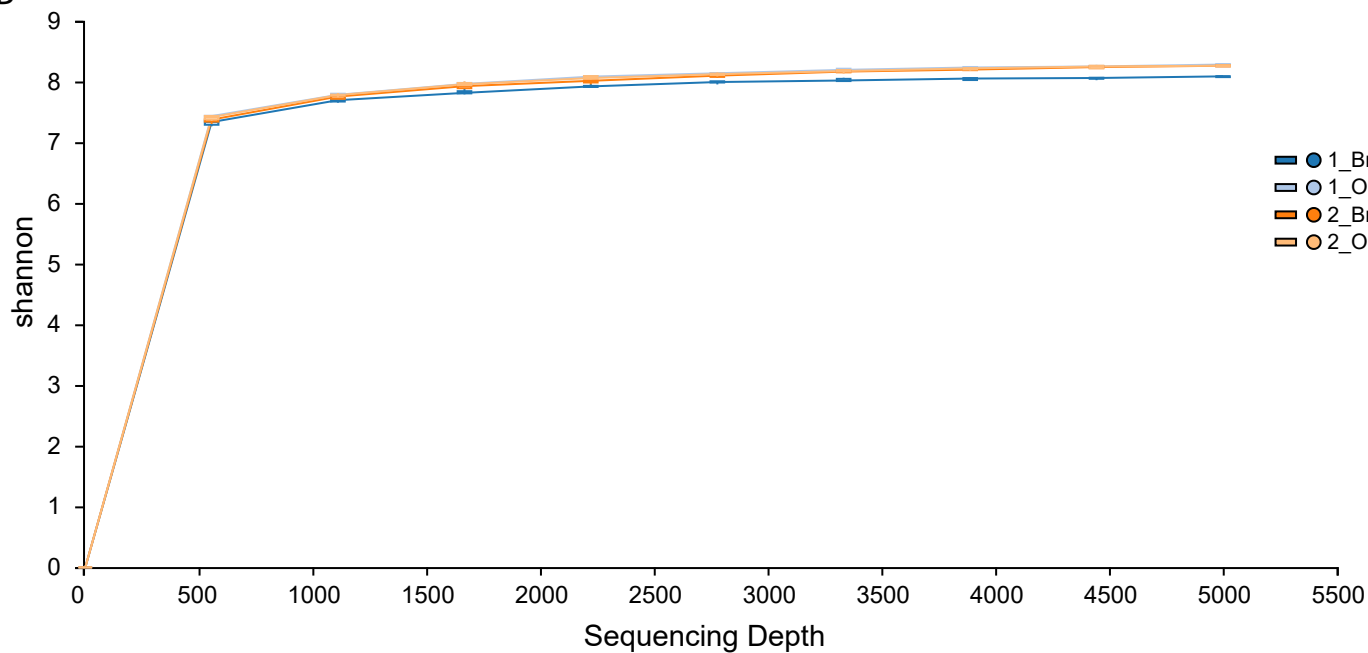

Supplement: Supplementary file 2 — Additional file 2: Figure S2. Rarefaction curve. (A) Rarefaction plot of vaginal samples (one curve per group). (B) Rarefaction plot of fecal samples. 1, 2, 3 and 4 in figure legend represent the pregnancy stage of pre-breeding, first trimester, second trimester, and third trimester, respectively. [file 40104_2019_401_MOESM2_ESM.pdf]

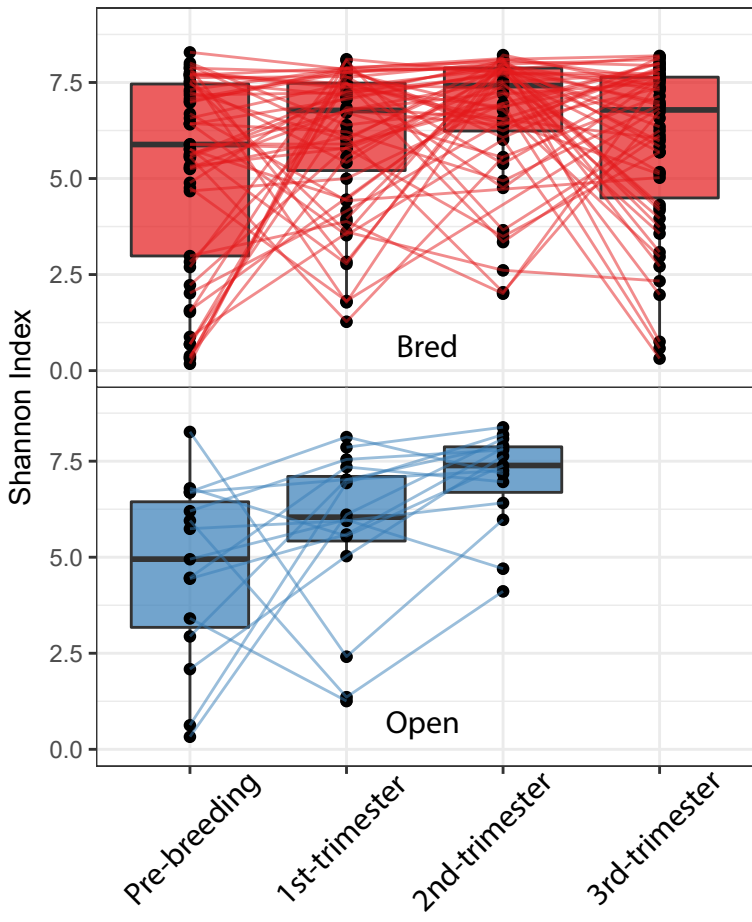

Supplement: Supplementary file 3 — Additional file 3: Figure S3. Alpha diversity (Shannon index) change of vaginal microbiota for each sample. The bottom and top of each box are the first and third quartiles, respectively, and the band inside the box is the median. The connected point represent alpha diversity of animal vaginal samples at different stage. [file 40104_2019_401_MOESM3_ESM.pdf]

**A**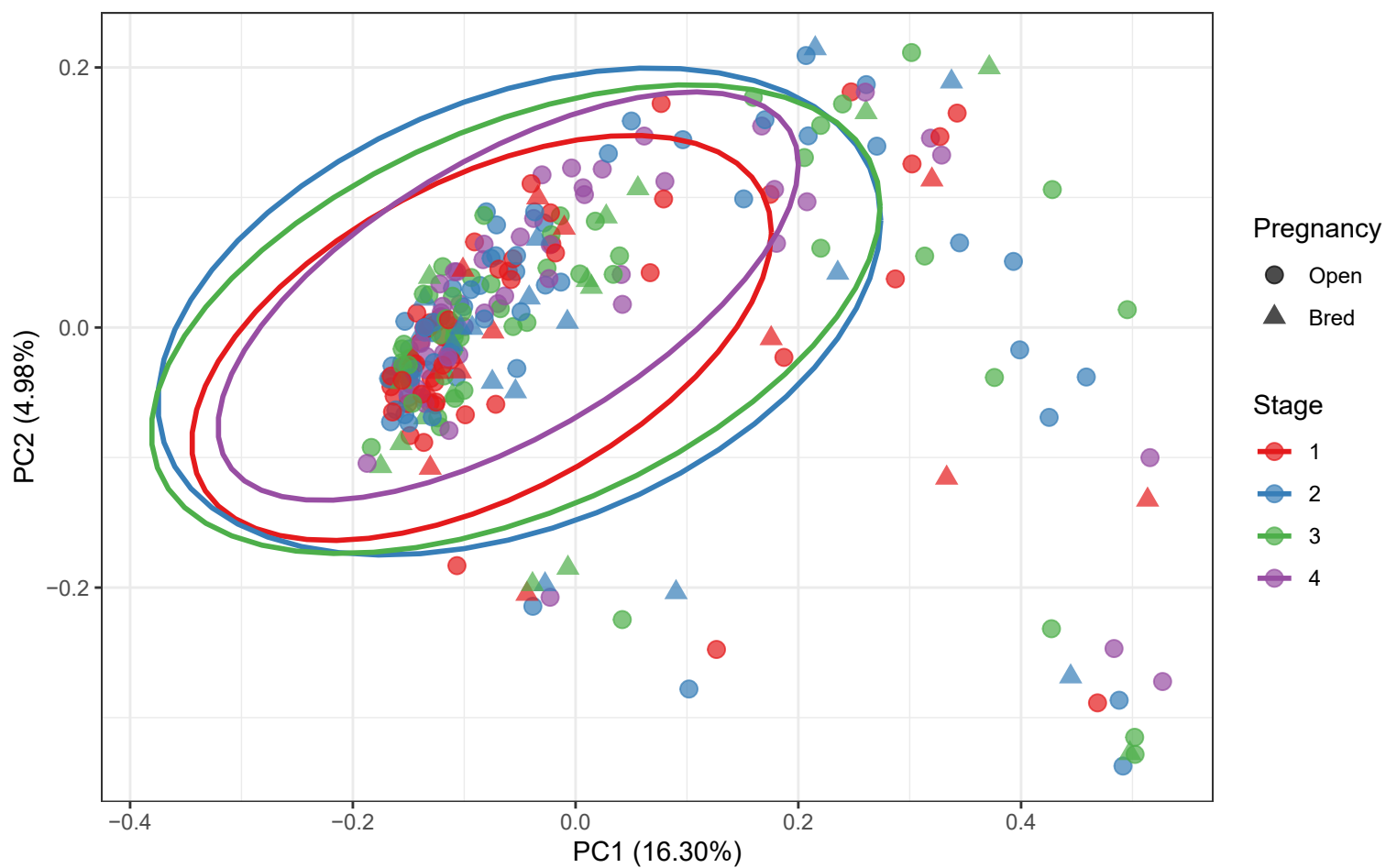**B**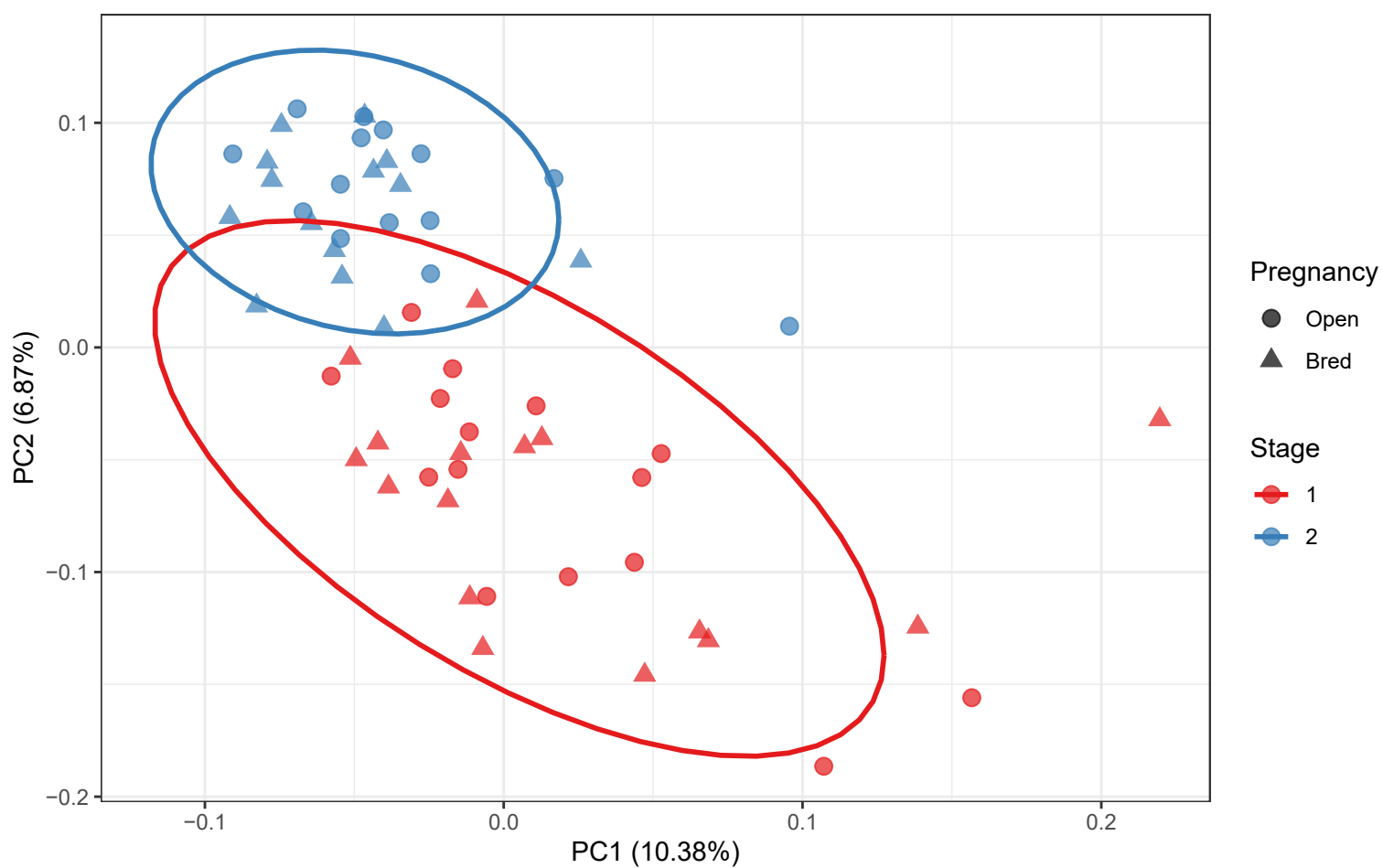

Supplement: Supplementary file 4 — Additional file 4: Figure S4. Principal Coordinates Analysis (PCoA) of unweighted UniFrac distances in vaginal (A) and fecal (B) samples across gestation stages and between open and bred cattle. Triangles and circle represent bred and open females, respectively. Stages are indicated by color: red, blue, green and purple represent pre breeding, and gestational trimesters 1 through 3 respectively. These stages were defined based on the status of the pregnant cattle. Open cattle stayed open throughout the whole experiment. Samples were collected prospectively but pregnancy was defined retrospectively. The ellipses were calculated and drawn with 0.95 of confidence level. Bred: cattle that were pregnant after the breeding season; Open: cattle that never established pregnancy. [file 40104_2019_401_MOESM4_ESM.pdf]

A

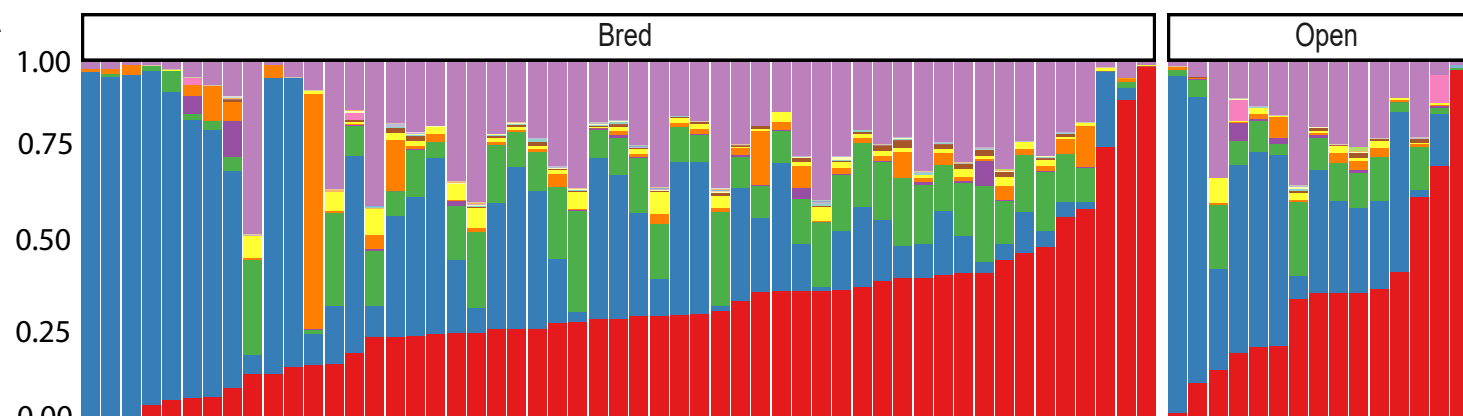

B

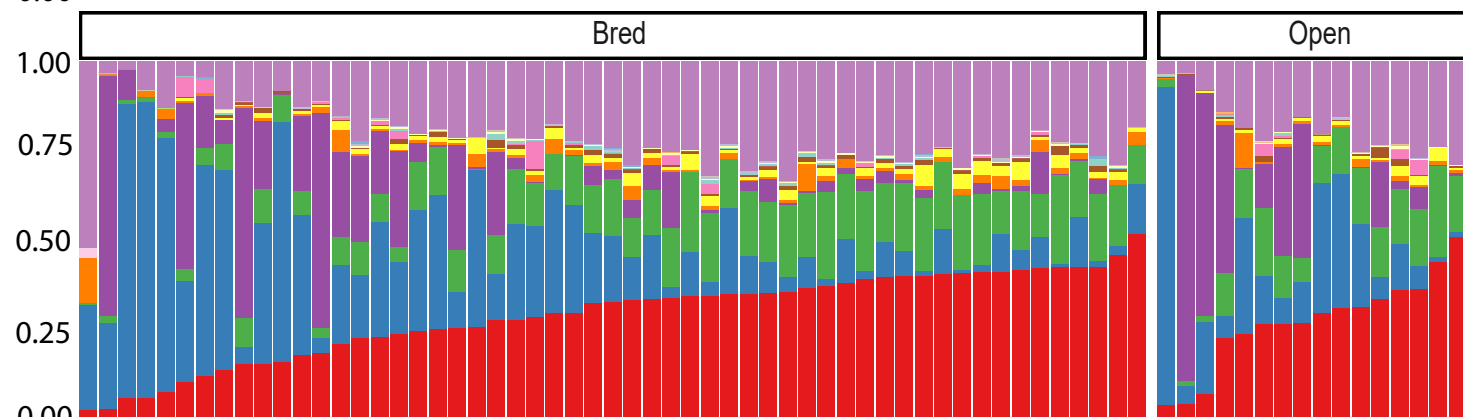

C

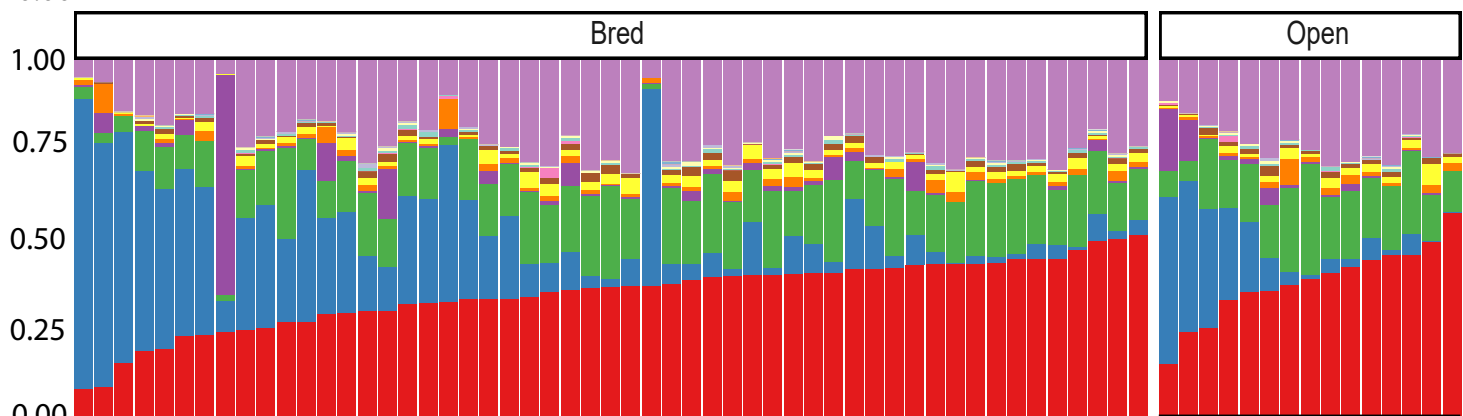

D

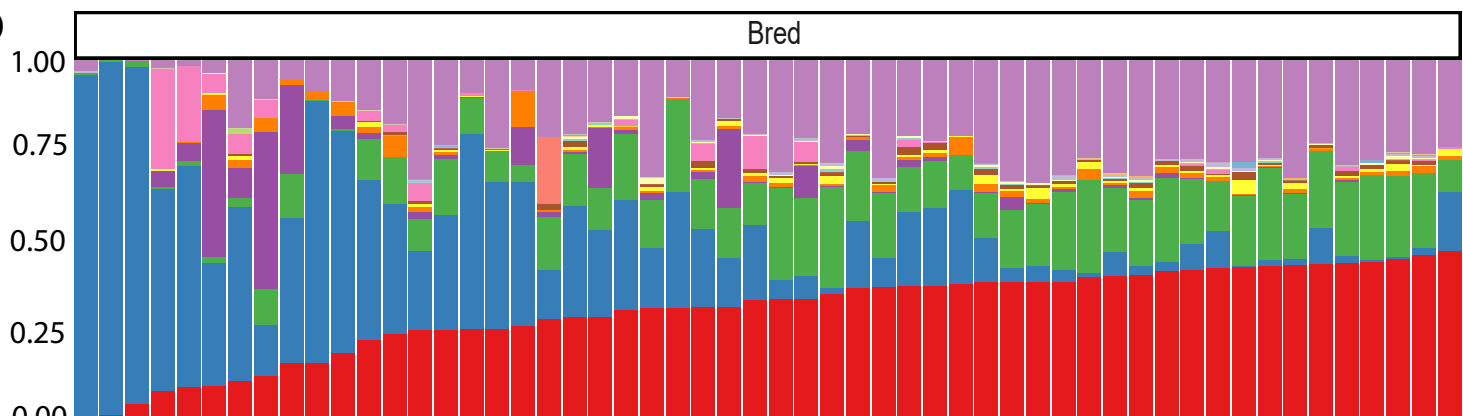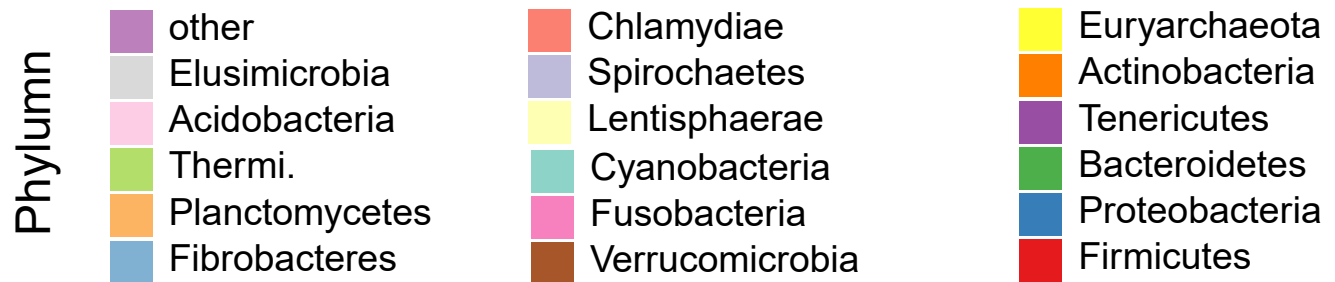

Supplement: Supplementary file 5 — Additional file 5: Figure S5. Relative abundance of vaginal microbiota at phylum level for each sample with different pregnancy status. Multi-colored stack bar graphs represent the relative abundance. Each chart represents a stage (A: Pre-breeding, B: first trimester, C: second trimester, D: third trimester). [file 40104_2019_401_MOESM5_ESM.pdf]

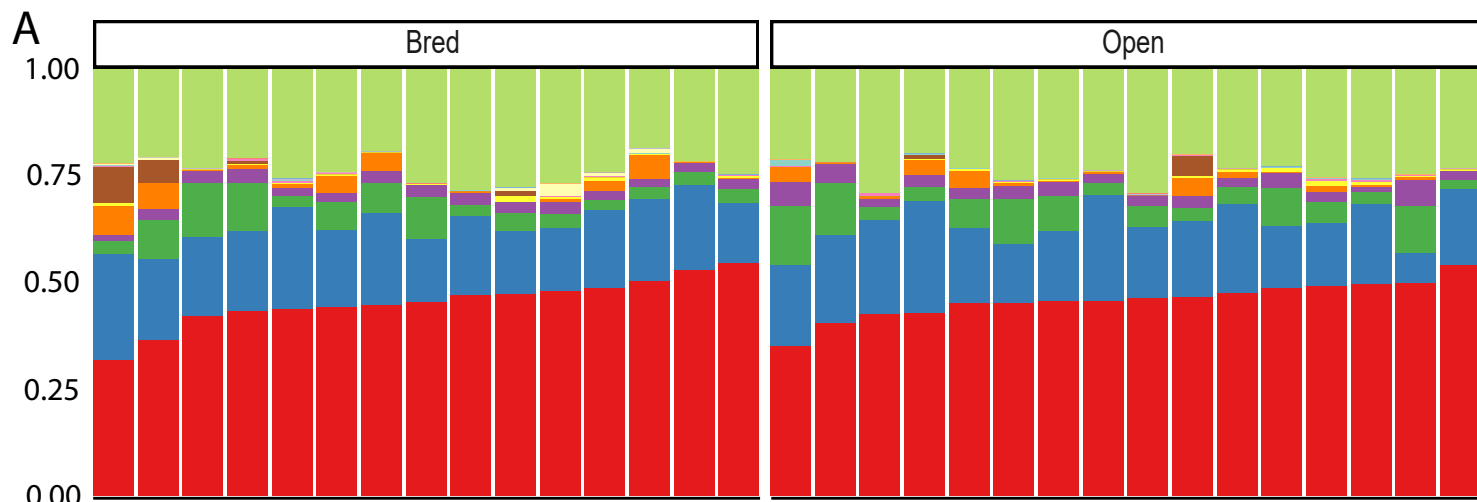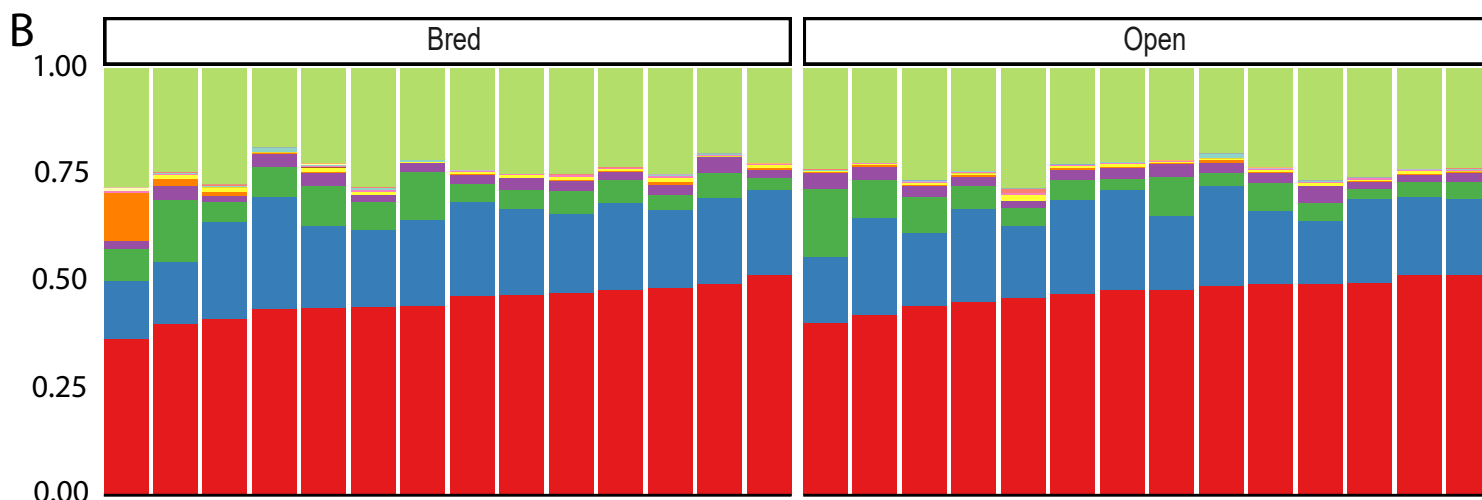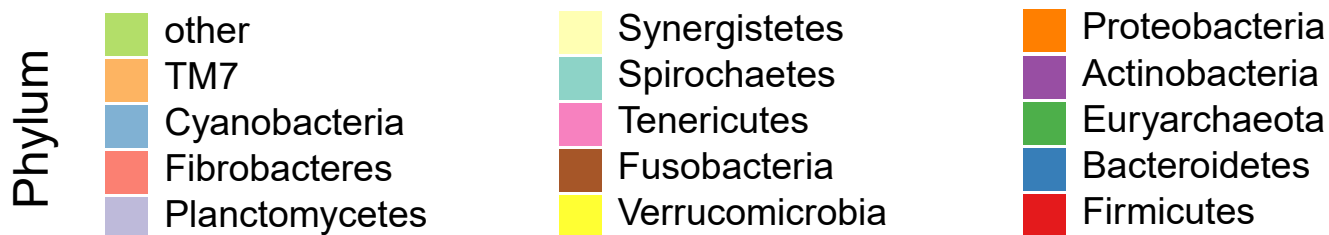

Supplement: Supplementary file 6 — Additional file 6: Figure S6. Relative abundance of fecal microbiota at phylum level for each sample with different pregnancy status. Multi-colored stack bar graphs represent the relative abundance. Each chart represents a stage (A: Pre-breeding, B: first trimester, C: second trimester, D: third trimester). [file 40104_2019_401_MOESM6_ESM.pdf]
