# Supplementary material for: The vaginal and fecal microbiomes are related to pregnancy status in beef heifers
Source: J Anim Sci Biotechnol. 2019 Dec 13;10:92. doi: 10.1186/s40104-019-0401-2 (PMC6909518; doi:10.1186/s40104-019-0401-2)
Supplement: Supplementary file 7 — Additional file 7: Figure S7. Relative abundance of predictive bacterial features in vaginal samples across gestation stages and between open and bred cattle. 1, 2, 3 and 4 On the X-axis represent the pregnancy stage of pre-breeding, first trimester, second trimester, and third trimester, respectively. [file 40104_2019_401_MOESM7_ESM.pdf]

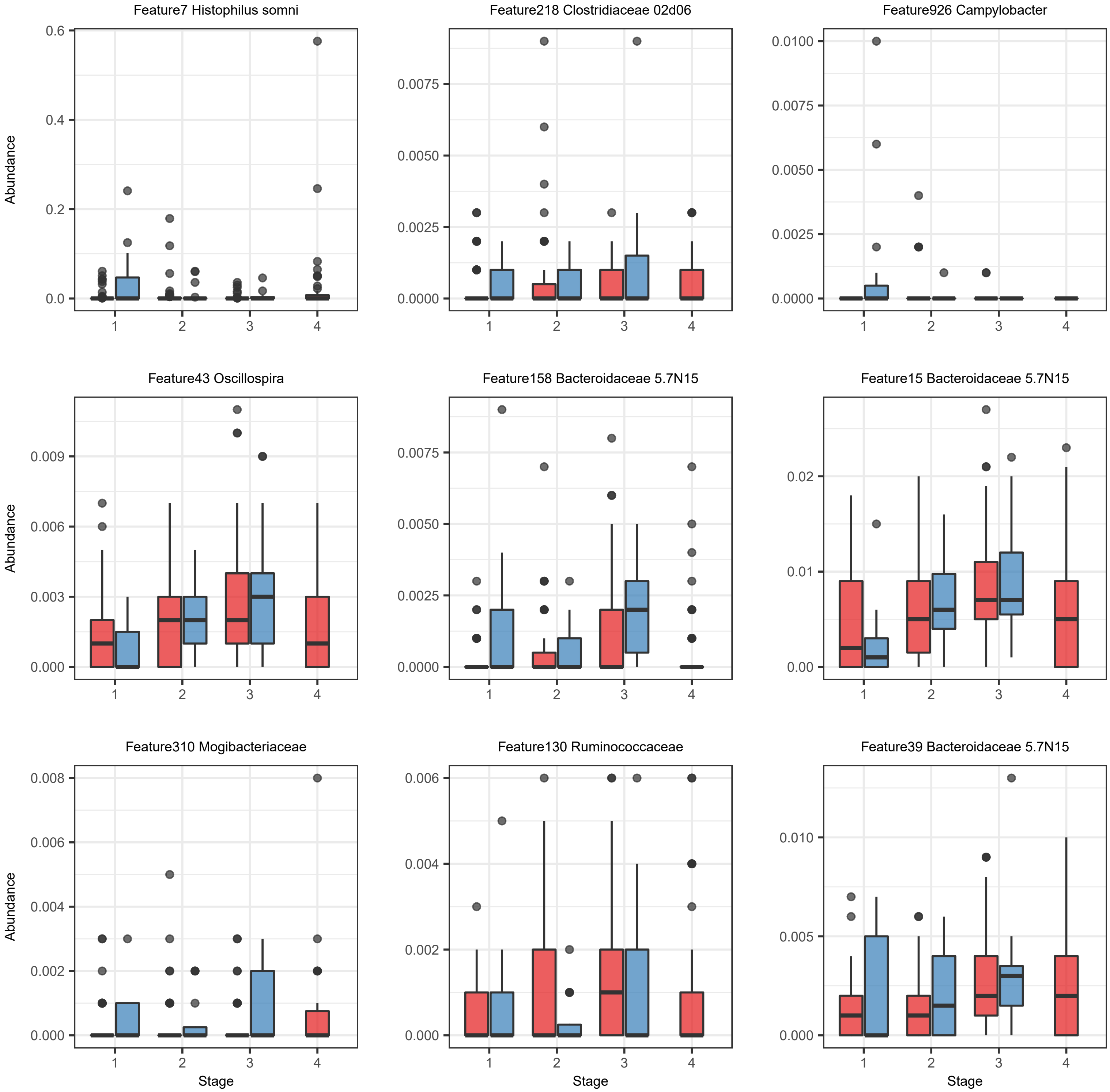
Feature7 Histophilus somni
Feature218 Clostridiaceae 02d06
Feature926 Campylobacter
Feature43 Oscillospira
Feature158 Bacteroidaceae 5.7N15
Feature15 Bacteroidaceae 5.7N15
Feature310 Mogibacteriaceae
Feature130 Ruminococcaceae
Feature39 Bacteroidaceae 5.7N15
Abundance
Stage
1
2
3
4
