# Supplementary material for: The vaginal and fecal microbiomes are related to pregnancy status in beef heifers
Source: J Anim Sci Biotechnol. 2019 Dec 13;10:92. doi: 10.1186/s40104-019-0401-2 (PMC6909518; doi:10.1186/s40104-019-0401-2)
Supplement: Supplementary file 8 — Additional file 8: Figure S8. Relative abundance of predictive bacterial features in fecal samples at pre-breeding and first trimester and between open and bred cattle. 1, 2, 3 and 4 On the X-axis represent the pregnancy stage of pre-breeding, first trimester, second trimester, and third trimester, respectively. [file 40104_2019_401_MOESM8_ESM.pdf]

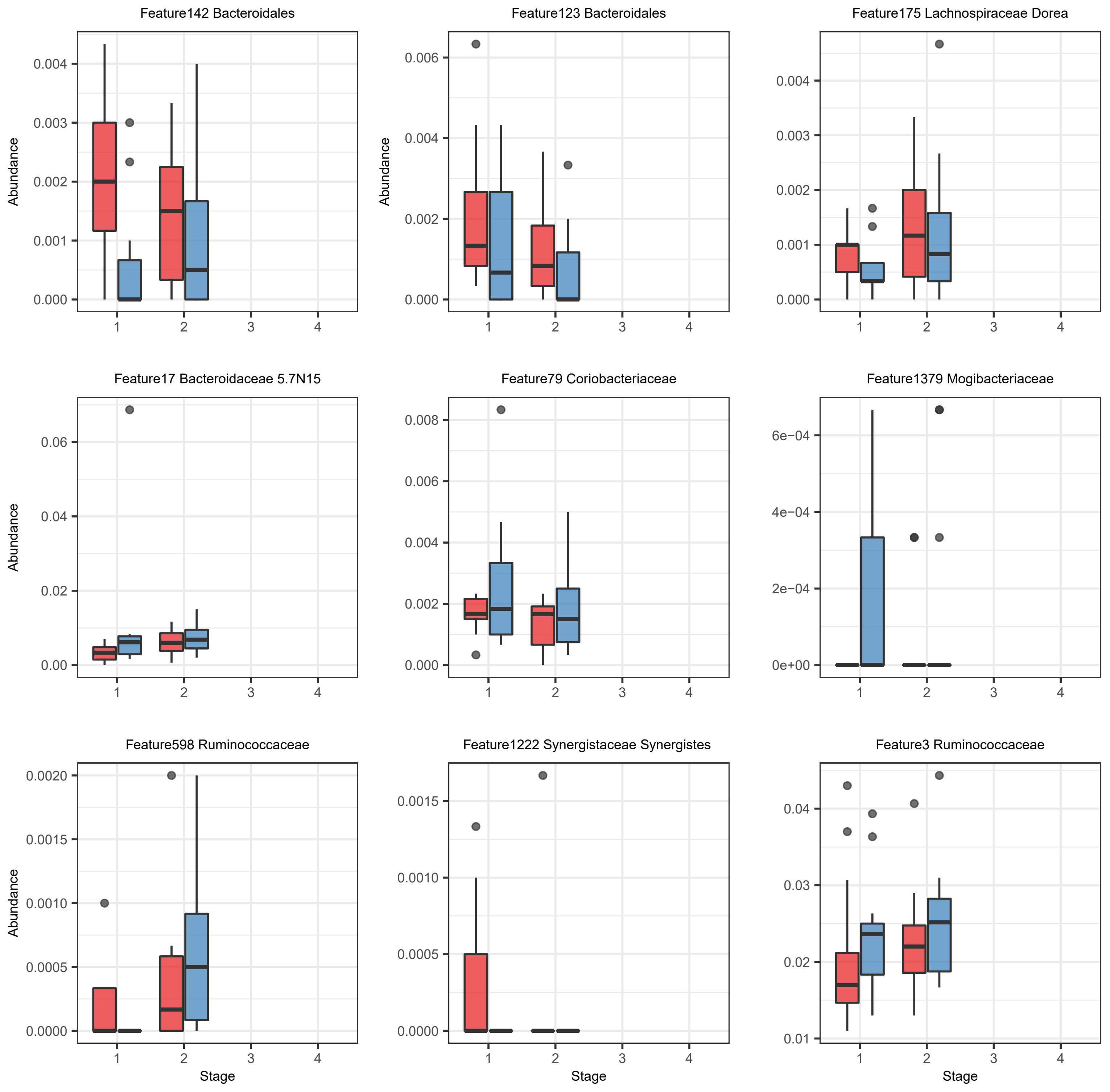

Feature142 Bacteroidales
Feature123 Bacteroidales
Feature175 Lachnospiraceae Dorea
Feature17 Bacteroidaceae 5.7N15
Feature79 Coriobacteriaceae
Feature1379 Mogibacteriaceae
Feature598 Ruminococcaceae
Feature1222 Synergistaceae Synergistes
Feature3 Ruminococcaceae
Abundance
Stage
1
2
3
4
