# Supplementary material for: The vaginal and fecal microbiomes are related to pregnancy status in beef heifers
Source: J Anim Sci Biotechnol. 2019 Dec 13;10:92. doi: 10.1186/s40104-019-0401-2 (PMC6909518; doi:10.1186/s40104-019-0401-2)
Supplement: Supplementary file 9 — Additional file 9: Table S1. Sequencing results of mock community. [file 40104_2019_401_MOESM9_ESM.docx]

**Table S1** Sequencing results of mock community

| Species | Expected  Abundance^*^ | Feature  Number | Reads  Count | Abundance |
| --- | --- | --- | --- | --- |
| Pseudomonas aeruginosa | 8.2% | 1 | 806 | 8.5% |
| Escherichia coli | 14.3% | 1 | 1918 | 20.2% |
| Salmonella enterica | 14.3% | 2 | 1620 | 17.1% |
| Lactobacillus fermentum | 10.2% | 1 | 667 | 7.0% |
| Enterococcus faecalis | 8.2% | 1 | 694 | 7.3% |
| Staphylococcus aureus | 12.2% | 1 | 1582 | 16.7% |
| Listeria monocytogenes | 12.2% | 1 | 707 | 7.5% |
| Bacillus subtilis | 20.4% | 1 | 1478 | 15.6% |
| Saccharomyces cerevisiae | 0 | 0 | 0 | 0 |
| Cryptococcus neoformans | 0 | 0 | 0 | 0 |

^*^ Normalized relative abundance with 16s rRNA gene copy number
