# Supplementary material for: The vaginal and fecal microbiomes are related to pregnancy status in beef heifers
Source: J Anim Sci Biotechnol. 2019 Dec 13;10:92. doi: 10.1186/s40104-019-0401-2 (PMC6909518; doi:10.1186/s40104-019-0401-2)
Supplement: Supplementary file 10 — Additional file 10: Table S2. Summary of sequencing and alpha diversity results for fecal and vaginal samples. [file 40104_2019_401_MOESM10_ESM.docx]

**Table S2** Summary of sequencing and alpha diversity results for fecal and vaginal samples

| Sample ID | Source | No. of raw reads | No. of clean reads | Observed OTUs | Shannon | Simpson |
| --- | --- | --- | --- | --- | --- | --- |
| 3 | Feces | 59,780 | 24,167 | 764 | 8.2241 | 0.9905 |
| 15 | Feces | 22,056 | 9,018 | 672 | 7.8241 | 0.9861 |
| 31 | Feces | 47,645 | 21,546 | 795 | 8.4259 | 0.9926 |
| 39 | Feces | 26,140 | 10,971 | 782 | 8.3396 | 0.991 |
| 47 | Feces | 21,482 | 9,952 | 633 | 7.6073 | 0.9822 |
| 63 | Feces | 27,706 | 12,811 | 681 | 7.7173 | 0.9847 |
| 75 | Feces | 13,119 | 5,745 | 688 | 8.2321 | 0.9907 |
| 83 | Feces | 19,623 | 8,130 | 595 | 7.7011 | 0.9869 |
| 87 | Feces | 20,760 | 9,245 | 738 | 8.2843 | 0.9919 |
| 91 | Feces | 23,776 | 10,115 | 629 | 7.6549 | 0.9849 |
| 111 | Feces | 25,644 | 11,808 | 683 | 8.0479 | 0.9902 |
| 127 | Feces | 22,643 | 9,957 | 726 | 8.3205 | 0.9922 |
| 139 | Feces | 18,202 | 7,654 | 683 | 7.9417 | 0.9861 |
| 151 | Feces | 21,605 | 9,104 | 735 | 8.1251 | 0.9896 |
| 159 | Feces | 24,222 | 9,823 | 629 | 7.8475 | 0.9891 |
| 163 | Feces | 19,003 | 7,230 | 544 | 7.4982 | 0.985 |
| 175 | Feces | 19,969 | 7,950 | 773 | 8.3521 | 0.9911 |
| 187 | Feces | 14,502 | 6,697 | 563 | 7.966 | 0.9908 |
| 199 | Feces | 16,073 | 6,059 | 645 | 8.1204 | 0.9901 |
| 207 | Feces | 30,389 | 13,255 | 648 | 7.8203 | 0.9862 |
| 215 | Feces | 28,787 | 12,671 | 786 | 8.4291 | 0.9922 |
| 219 | Feces | 35,363 | 15,562 | 845 | 8.5326 | 0.9922 |
| 227 | Feces | 33,864 | 12,398 | 766 | 8.2388 | 0.9902 |
| 231 | Feces | 27,655 | 10,914 | 714 | 8.1162 | 0.9902 |
| 235 | Feces | 28,833 | 10,418 | 712 | 7.9623 | 0.987 |
| 239 | Feces | 31,201 | 11,823 | 769 | 8.2219 | 0.9898 |
| 247 | Feces | 20,109 | 8,960 | 677 | 8.1203 | 0.9906 |
| 259 | Feces | 24,881 | 8,946 | 385 | 6.9276 | 0.9774 |
| 263 | Feces | 27,018 | 10,890 | 761 | 8.255 | 0.9884 |
| 267 | Feces | 18,175 | 6,268 | 724 | 8.1633 | 0.9879 |
| 275 | Feces | 35,261 | 13,094 | 674 | 7.7485 | 0.9842 |
| 303 | Feces | 8,372 | 3,045 | 489 | 7.6085 | 0.9827 |
| 327 | Feces | 33,909 | 15,295 | 799 | 8.4408 | 0.9929 |
| 335 | Feces | 24,955 | 11,758 | 726 | 8.2584 | 0.9907 |
| 351 | Feces | 30,908 | 13,829 | 724 | 8.1392 | 0.9901 |
| 363 | Feces | 103,205 | 44,271 | 753 | 8.1616 | 0.9904 |
| 371 | Feces | 26,008 | 9,976 | 716 | 7.998 | 0.9849 |
| 375 | Feces | 28,338 | 11,552 | 706 | 7.9404 | 0.9868 |
| 379 | Feces | 64,504 | 38,109 | 588 | 7.7847 | 0.9835 |
| 415 | Feces | 221,759 | 80,428 | 761 | 7.8689 | 0.9809 |
| 427 | Feces | 108,761 | 39,196 | 801 | 8.269 | 0.9891 |
| 439 | Feces | 64,479 | 26,256 | 769 | 8.1995 | 0.9899 |
| 447 | Feces | 38,792 | 16,604 | 785 | 8.314 | 0.9906 |
| 451 | Feces | 29,867 | 12,510 | 695 | 7.8083 | 0.9841 |
| 463 | Feces | 26,969 | 12,120 | 744 | 8.2932 | 0.9919 |
| 475 | Feces | 328,769 | 131,320 | 761 | 8.1632 | 0.987 |
| 487 | Feces | 24,949 | 12,106 | 759 | 8.4172 | 0.9928 |
| 495 | Feces | 1,166,963 | 453,279 | 674 | 7.7245 | 0.9803 |
| 503 | Feces | 29,764 | 11,366 | 736 | 8.197 | 0.9875 |
| 507 | Feces | 47,574 | 20,430 | 803 | 8.3647 | 0.9903 |
| 515 | Feces | 73,727 | 29,510 | 647 | 7.7798 | 0.9865 |
| 519 | Feces | 30,681 | 12,624 | 785 | 8.3006 | 0.989 |
| 523 | Feces | 101,050 | 43,543 | 750 | 8.0495 | 0.9865 |
| 527 | Feces | 63,951 | 28,874 | 807 | 8.4793 | 0.993 |
| 547 | Feces | 16,190 | 7,228 | 680 | 8.1752 | 0.9912 |
| 551 | Feces | 30,337 | 13,634 | 748 | 7.9526 | 0.9824 |
| 555 | Feces | 96,399 | 42,520 | 821 | 8.4407 | 0.9919 |
| 563 | Feces | 16,425 | 7,483 | 635 | 7.9988 | 0.9883 |
| 567 | Feces | 22,612 | 8,823 | 692 | 8.0671 | 0.9883 |
| 4 | vagina | 34,062 | 15,138 | 267 | 0.9136 | 5.9556 |
| 8 | vagina | 32,381 | 14,536 | 399 | 0.9872 | 7.6258 |
| 12 | vagina | 21,199 | 14,907 | 184 | 0.9037 | 5.5648 |
| 16 | vagina | 32,963 | 12,574 | 458 | 0.9753 | 7.6829 |
| 20 | vagina | 52,675 | 25,285 | 395 | 0.9817 | 7.5067 |
| 24 | vagina | 49,759 | 20,378 | 421 | 0.988 | 7.72 |
| 28 | vagina | 30,721 | 13,686 | 369 | 0.9711 | 7.0141 |
| 32 | vagina | 44,945 | 18,452 | 322 | 0.9736 | 6.9816 |
| 36 | vagina | 29,619 | 12,524 | 492 | 0.9937 | 8.2861 |
| 40 | vagina | 74,489 | 36,114 | 498 | 0.9933 | 8.2641 |
| 44 | vagina | 39,811 | 16,058 | 391 | 0.9823 | 7.4602 |
| 48 | vagina | 31,234 | 12,641 | 412 | 0.9862 | 7.6957 |
| 52 | vagina | 3,069 | 1,731 | 9 | 0.562 | 1.574 |
| 56 | vagina | 125,008 | 66,846 | 427 | 0.988 | 7.7671 |
| 60 | vagina | 5,473 | 3,898 | 10 | 0.0356 | 0.1801 |
| 64 | vagina | 5,734 | 3,998 | 11 | 0.0685 | 0.3128 |
| 68 | vagina | 3,295 | 2,319 | 14 | 0.2935 | 0.8804 |
| 72 | vagina | 3,775 | 2,492 | 8 | 0.0924 | 0.345 |
| 84 | vagina | 2,231 | 1,495 | 8 | 0.2074 | 0.6279 |
| 96 | vagina | 1,838 | 1,153 | 7 | 0.2476 | 0.6887 |
| 100 | vagina | 35,422 | 22,932 | 84 | 0.6155 | 2.8326 |
| 104 | vagina | 38,530 | 21,196 | 357 | 0.9252 | 6.4129 |
| 108 | vagina | 40,745 | 24,861 | 310 | 0.8039 | 5.3224 |
| 112 | vagina | 36,405 | 22,924 | 284 | 0.8524 | 5.6039 |
| 116 | vagina | 32,636 | 20,642 | 252 | 0.7618 | 4.6745 |
| 120 | vagina | 16,731 | 9,237 | 336 | 0.9163 | 6.4366 |
| 124 | vagina | 20,765 | 14,529 | 53 | 0.5501 | 2.2263 |
| 128 | vagina | 21,897 | 14,442 | 256 | 0.9581 | 6.4343 |
| 132 | vagina | 25,464 | 16,525 | 171 | 0.8817 | 5.2745 |
| 136 | vagina | 25,925 | 14,937 | 338 | 0.8516 | 5.7708 |
| 140 | vagina | 14,385 | 9,747 | 54 | 0.4349 | 2.0179 |
| 144 | vagina | 18,362 | 11,612 | 214 | 0.7944 | 4.8915 |
| 148 | vagina | 10,089 | 5,436 | 254 | 0.8738 | 5.637 |
| 152 | vagina | 14,168 | 8,049 | 304 | 0.8738 | 5.7583 |
| 156 | vagina | 17,522 | 9,068 | 287 | 0.8163 | 5.2514 |
| 160 | vagina | 11,670 | 7,253 | 239 | 0.9353 | 6.2011 |
| 164 | vagina | 11,261 | 7,260 | 136 | 0.8157 | 4.4674 |
| 168 | vagina | 47,667 | 26,109 | 395 | 0.9706 | 7.3093 |
| 172 | vagina | 16,703 | 8,696 | 318 | 0.945 | 6.574 |
| 176 | vagina | 19,711 | 11,688 | 380 | 0.9686 | 7.2483 |
| 180 | vagina | 29,819 | 15,508 | 373 | 0.9652 | 7.0784 |
| 184 | vagina | 16,009 | 9,345 | 421 | 0.9909 | 7.8735 |
| 188 | vagina | 44,601 | 20,388 | 330 | 0.9812 | 7.1766 |
| 192 | vagina | 31,532 | 15,278 | 439 | 0.9908 | 7.9476 |
| 196 | vagina | 78,532 | 43,776 | 446 | 0.9889 | 7.8774 |
| 200 | vagina | 24,872 | 15,891 | 124 | 0.3735 | 2.0891 |
| 204 | vagina | 16,852 | 11,061 | 57 | 0.3314 | 1.5327 |
| 208 | vagina | 16,221 | 10,754 | 39 | 0.1383 | 0.691 |
| 212 | vagina | 16,645 | 11,511 | 15 | 0.0801 | 0.3867 |
| 216 | vagina | 17,963 | 12,331 | 18 | 0.0648 | 0.3289 |
| 220 | vagina | 13,958 | 8,227 | 80 | 0.7472 | 2.9411 |
| 224 | vagina | 11,950 | 6,104 | 97 | 0.6536 | 2.6974 |
| 228 | vagina | 40,873 | 18,123 | 343 | 0.9652 | 6.7965 |
| 232 | vagina | 24,218 | 16,375 | 264 | 0.9587 | 6.7046 |
| 236 | vagina | 31,340 | 16,101 | 287 | 0.8649 | 5.549 |
| 240 | vagina | 78,890 | 32,910 | 360 | 0.9357 | 6.6852 |
| 244 | vagina | 40,264 | 21,647 | 453 | 0.9903 | 8.0091 |
| 248 | vagina | 25,681 | 15,365 | 269 | 0.9124 | 5.7424 |
| 252 | vagina | 20,031 | 11,804 | 306 | 0.9217 | 5.8942 |
| 256 | vagina | 14,798 | 8,204 | 337 | 0.9563 | 6.6881 |
| 260 | vagina | 16,347 | 11,015 | 82 | 0.8017 | 3.4083 |
| 264 | vagina | 13,945 | 9,629 | 181 | 0.8086 | 4.4429 |
| 268 | vagina | 16,734 | 11,919 | 89 | 0.6293 | 2.9843 |
| 272 | vagina | 15,688 | 10,591 | 178 | 0.8006 | 4.7578 |
| 276 | vagina | 30,087 | 19,649 | 253 | 0.8478 | 4.9501 |
| 280 | vagina | 11,726 | 6,078 | 391 | 0.988 | 7.7098 |
| 284 | vagina | 36,909 | 25,051 | 206 | 0.9375 | 5.885 |
| 288 | vagina | 47,868 | 29,486 | 389 | 0.9849 | 7.5385 |
| 292 | vagina | 11,651 | 8,571 | 64 | 0.2817 | 1.3589 |
| 296 | vagina | 12,392 | 5,700 | 372 | 0.9887 | 7.6529 |
| 300 | vagina | 2,772 | 1,877 | 19 | 0.3256 | 1.2733 |
| 304 | vagina | 11,353 | 4,947 | 416 | 0.9893 | 7.8767 |
| 308 | vagina | 11,012 | 7,219 | 173 | 0.6429 | 3.611 |
| 312 | vagina | 9,891 | 4,387 | 343 | 0.9882 | 7.4656 |
| 316 | vagina | 10,299 | 5,422 | 393 | 0.99 | 7.758 |
| 320 | vagina | 13,332 | 7,370 | 408 | 0.9894 | 7.8668 |
| 324 | vagina | 10,807 | 4,839 | 400 | 0.9901 | 7.842 |
| 328 | vagina | 15,046 | 10,699 | 79 | 0.5749 | 2.4123 |
| 332 | vagina | 18,096 | 11,647 | 177 | 0.8821 | 5.4161 |
| 336 | vagina | 19,730 | 13,981 | 45 | 0.5122 | 1.7798 |
| 344 | vagina | 16,555 | 9,064 | 404 | 0.9711 | 7.3873 |
| 348 | vagina | 12,750 | 6,257 | 459 | 0.9918 | 8.1079 |
| 352 | vagina | 15,813 | 7,577 | 409 | 0.9914 | 7.8873 |
| 356 | vagina | 11,303 | 7,958 | 91 | 0.6877 | 3.5783 |
| 360 | vagina | 9,099 | 4,851 | 389 | 0.9859 | 7.6293 |
| 364 | vagina | 4,992 | 2,798 | 330 | 0.9854 | 7.4575 |
| 368 | vagina | 11,743 | 7,981 | 215 | 0.6603 | 3.953 |
| 372 | vagina | 18,565 | 11,635 | 333 | 0.9726 | 6.9345 |
| 376 | vagina | 22,483 | 11,932 | 430 | 0.9896 | 7.8632 |
| 380 | vagina | 15,881 | 10,211 | 308 | 0.9443 | 6.7736 |
| 384 | vagina | 34,514 | 19,801 | 356 | 0.9552 | 6.7171 |
| 388 | vagina | 15,668 | 10,073 | 302 | 0.9613 | 6.8466 |
| 392 | vagina | 34,075 | 20,778 | 357 | 0.9762 | 7.0625 |
| 396 | vagina | 30,148 | 15,309 | 314 | 0.8661 | 5.7894 |
| 400 | vagina | 22,713 | 14,018 | 321 | 0.9807 | 7.268 |
| 404 | vagina | 10,648 | 7,497 | 162 | 0.8743 | 4.9983 |
| 408 | vagina | 11,241 | 7,587 | 103 | 0.7861 | 3.5247 |
| 412 | vagina | 6,535 | 4,114 | 274 | 0.9116 | 5.8212 |
| 416 | vagina | 7,974 | 4,817 | 257 | 0.9446 | 6.2162 |
| 420 | vagina | 11,437 | 6,239 | 323 | 0.9624 | 6.773 |
| 424 | vagina | 7,803 | 5,320 | 39 | 0.6289 | 2.7789 |
| 428 | vagina | 9,927 | 7,293 | 99 | 0.8174 | 4.1549 |
| 432 | vagina | 16,789 | 11,167 | 321 | 0.9844 | 7.4622 |
| 436 | vagina | 13,538 | 9,093 | 245 | 0.9737 | 6.7671 |
| 440 | vagina | 15,010 | 11,088 | 110 | 0.8713 | 4.4543 |
| 444 | vagina | 10,318 | 6,895 | 153 | 0.9504 | 6.0826 |
| 448 | vagina | 13,379 | 8,198 | 358 | 0.9875 | 7.5456 |
| 452 | vagina | 6,420 | 3,459 | 339 | 0.9831 | 7.3499 |
| 456 | vagina | 10,480 | 5,930 | 411 | 0.9905 | 7.861 |
| 460 | vagina | 11,821 | 7,185 | 347 | 0.9834 | 7.4726 |
| 464 | vagina | 6,441 | 3,502 | 226 | 0.9284 | 6.0001 |
| 468 | vagina | 16,985 | 9,043 | 405 | 0.9892 | 7.7993 |
| 472 | vagina | 11,802 | 6,990 | 396 | 0.9883 | 7.7607 |
| 476 | vagina | 30,251 | 19,779 | 365 | 0.9649 | 7.2325 |
| 480 | vagina | 18,649 | 12,292 | 238 | 0.9425 | 6.0557 |
| 484 | vagina | 9,426 | 6,754 | 80 | 0.5548 | 2.837 |
| 488 | vagina | 18,414 | 12,921 | 198 | 0.8524 | 5.0315 |
| 492 | vagina | 37,471 | 19,384 | 417 | 0.9887 | 7.7586 |
| 496 | vagina | 13,433 | 7,125 | 393 | 0.9778 | 7.4944 |
| 500 | vagina | 4,920 | 3,320 | 217 | 0.9064 | 5.5602 |
| 504 | vagina | 21,542 | 14,486 | 304 | 0.9014 | 6.1101 |
| 508 | vagina | 12,959 | 7,879 | 364 | 0.9603 | 7.0195 |
| 512 | vagina | 35,547 | 19,413 | 440 | 0.9924 | 8.0779 |
| 516 | vagina | 47,875 | 30,429 | 235 | 0.9059 | 5.5512 |
| 520 | vagina | 31,647 | 15,441 | 463 | 0.9922 | 8.1303 |
| 524 | vagina | 77,247 | 36,460 | 378 | 0.9719 | 7.1094 |
| 528 | vagina | 25,959 | 15,296 | 396 | 0.9488 | 7.021 |
| 532 | vagina | 33,764 | 19,172 | 356 | 0.9118 | 6.3936 |
| 536 | vagina | 18,853 | 11,185 | 335 | 0.8695 | 5.9504 |
| 540 | vagina | 18,431 | 11,853 | 298 | 0.8876 | 5.8971 |
| 544 | vagina | 7,277 | 4,477 | 157 | 0.8046 | 4.4213 |
| 548 | vagina | 5,881 | 3,986 | 78 | 0.2253 | 1.2579 |
| 552 | vagina | 16,687 | 9,117 | 286 | 0.8604 | 5.5947 |
| 556 | vagina | 13,524 | 9,659 | 127 | 0.8486 | 3.8825 |
| 560 | vagina | 5,408 | 3,536 | 97 | 0.3302 | 1.8143 |
| 564 | vagina | 13,636 | 7,680 | 298 | 0.8928 | 5.9751 |
| 568 | vagina | 14,418 | 9,059 | 324 | 0.9833 | 7.3596 |
| 572 | vagina | 22,778 | 14,636 | 269 | 0.9227 | 5.7803 |
| 576 | vagina | 20,089 | 11,612 | 388 | 0.9591 | 7.1628 |
| 580 | vagina | 19,397 | 13,584 | 179 | 0.7582 | 4.1132 |
| 584 | vagina | 16,447 | 10,253 | 314 | 0.8027 | 5.3868 |
| 588 | vagina | 16,814 | 8,938 | 319 | 0.8313 | 5.5602 |
| 592 | vagina | 11,090 | 6,897 | 276 | 0.9163 | 6.0627 |
| 596 | vagina | 6,545 | 4,121 | 113 | 0.5336 | 2.6118 |
| 600 | vagina | 8,890 | 4,790 | 332 | 0.9234 | 6.4273 |
| 604 | vagina | 23,604 | 12,802 | 348 | 0.9464 | 6.7781 |
| 608 | vagina | 16,520 | 7,814 | 456 | 0.9926 | 8.1262 |
| 612 | vagina | 17,603 | 9,346 | 340 | 0.9067 | 6.2363 |
| 616 | vagina | 26,030 | 14,375 | 302 | 0.8896 | 5.9777 |
| 620 | vagina | 10,632 | 6,091 | 399 | 0.9903 | 7.8492 |
| 624 | vagina | 17,324 | 11,093 | 388 | 0.9893 | 7.8719 |
| 628 | vagina | 15,952 | 10,572 | 314 | 0.9816 | 7.4202 |
| 632 | vagina | 14,688 | 7,977 | 406 | 0.9916 | 7.855 |
| 636 | vagina | 14,610 | 9,019 | 380 | 0.9896 | 7.7433 |
| 640 | vagina | 11,702 | 6,629 | 431 | 0.9924 | 8.0483 |
| 644 | vagina | 15,923 | 8,038 | 476 | 0.9927 | 8.2159 |
| 648 | vagina | 11,080 | 6,115 | 418 | 0.9924 | 8.0231 |
| 652 | vagina | 6,507 | 3,797 | 355 | 0.9805 | 7.3387 |
| 656 | vagina | 13,084 | 5,972 | 413 | 0.9925 | 7.9766 |
| 660 | vagina | 21,433 | 11,661 | 463 | 0.9931 | 8.1928 |
| 664 | vagina | 33,292 | 16,187 | 497 | 0.9941 | 8.3845 |
| 668 | vagina | 47,442 | 28,607 | 384 | 0.9775 | 7.5415 |
| 672 | vagina | 16,733 | 9,887 | 327 | 0.8212 | 5.5476 |
| 676 | vagina | 13,356 | 8,084 | 348 | 0.9198 | 6.558 |
| 680 | vagina | 10,511 | 5,640 | 428 | 0.9857 | 7.786 |
| 684 | vagina | 22,483 | 14,885 | 71 | 0.8649 | 3.4913 |
| 688 | vagina | 15,913 | 7,829 | 439 | 0.9902 | 7.9617 |
| 692 | vagina | 9,978 | 5,672 | 317 | 0.9771 | 6.9518 |
| 696 | vagina | 15,432 | 9,135 | 314 | 0.8857 | 6.0125 |
| 700 | vagina | 18,448 | 10,251 | 413 | 0.9739 | 7.5139 |
| 704 | vagina | 6,889 | 4,275 | 255 | 0.786 | 4.938 |
| 708 | vagina | 21,961 | 12,950 | 377 | 0.9665 | 7.2391 |
| 712 | vagina | 63,330 | 33,612 | 481 | 0.9889 | 8.0658 |
| 716 | vagina | 19,469 | 10,490 | 351 | 0.9155 | 6.4842 |
| 720 | vagina | 7,541 | 4,826 | 271 | 0.9562 | 6.5348 |
| 724 | vagina | 14,425 | 8,606 | 340 | 0.9079 | 6.389 |
| 728 | vagina | 11,297 | 6,112 | 405 | 0.9754 | 7.4821 |
| 732 | vagina | 11,403 | 8,227 | 60 | 0.5726 | 2.0416 |
| 736 | vagina | 10,319 | 5,345 | 412 | 0.9878 | 7.7925 |
| 740 | vagina | 16,414 | 8,447 | 356 | 0.9616 | 6.9623 |
| 744 | vagina | 13,804 | 7,681 | 398 | 0.9899 | 7.8149 |
| 748 | vagina | 20,806 | 13,913 | 234 | 0.7547 | 4.7582 |
| 752 | vagina | 21,950 | 10,470 | 447 | 0.9921 | 8.1095 |
| 756 | vagina | 302,558 | 141,217 | 427 | 0.9911 | 7.8823 |
| 760 | vagina | 29,807 | 14,392 | 456 | 0.9919 | 8.1021 |
| 764 | vagina | 19,564 | 9,832 | 400 | 0.9865 | 7.7007 |
| 768 | vagina | 17,169 | 8,224 | 423 | 0.9903 | 7.9014 |
| 772 | vagina | 29,356 | 16,601 | 421 | 0.9892 | 7.876 |
| 776 | vagina | 26,771 | 11,687 | 394 | 0.9879 | 7.635 |
| 780 | vagina | 18,934 | 10,556 | 422 | 0.9913 | 7.9555 |
| 784 | vagina | 28,656 | 20,645 | 97 | 0.6205 | 3.3481 |
| 788 | vagina | 67,664 | 39,081 | 412 | 0.9883 | 7.8248 |
| 792 | vagina | 198,267 | 98,623 | 451 | 0.9926 | 8.0859 |
| 796 | vagina | 25,663 | 14,558 | 443 | 0.9899 | 7.9063 |
| 800 | vagina | 7,262 | 4,106 | 91 | 0.7904 | 3.6564 |
| 804 | vagina | 16,862 | 7,224 | 372 | 0.9794 | 7.3879 |
| 808 | vagina | 39,023 | 14,970 | 362 | 0.9793 | 7.1712 |
| 812 | vagina | 52,736 | 25,908 | 427 | 0.9856 | 7.7406 |
| 816 | vagina | 13,392 | 7,301 | 403 | 0.9607 | 7.2571 |
| 820 | vagina | 4,080 | 2,166 | 297 | 0.9581 | 6.865 |
| 824 | vagina | 2,479 | 1,282 | 243 | 0.9406 | 6.4151 |
| 840 | vagina | 29,348 | 13,729 | 430 | 0.989 | 7.841 |
| 844 | vagina | 2,752 | 1,693 | 100 | 0.3626 | 2.005 |
| 852 | vagina | 28,308 | 19,298 | 176 | 0.8105 | 4.7032 |
| 856 | vagina | 67,798 | 44,341 | 339 | 0.9453 | 6.8633 |
| 860 | vagina | 16,097 | 10,533 | 351 | 0.9696 | 7.1995 |
| 864 | vagina | 49,919 | 27,787 | 454 | 0.9916 | 8.0609 |
| 868 | vagina | 50,341 | 23,452 | 417 | 0.9858 | 7.6705 |
| 872 | vagina | 48,479 | 32,100 | 313 | 0.9781 | 7.1499 |
| 876 | vagina | 25,611 | 13,213 | 403 | 0.9916 | 7.8409 |
| 880 | vagina | 32,287 | 24,481 | 47 | 0.6374 | 2.3324 |
| 884 | vagina | 92,456 | 51,077 | 386 | 0.9814 | 7.3552 |
| 888 | vagina | 10,941 | 7,606 | 42 | 0.6508 | 2.9647 |
| 892 | vagina | 52,254 | 37,132 | 29 | 0.162 | 0.7539 |
| 896 | vagina | 27,254 | 13,175 | 455 | 0.993 | 8.1551 |
| 900 | vagina | 16,784 | 12,349 | 61 | 0.5692 | 2.7151 |
| 904 | vagina | 55,609 | 39,146 | 213 | 0.9121 | 5.9774 |
| 908 | vagina | 27,898 | 20,187 | 181 | 0.8404 | 5.1698 |
| 912 | vagina | 2,616 | 1,330 | 252 | 0.9757 | 6.9897 |
| 916 | vagina | 27,695 | 17,859 | 361 | 0.9889 | 7.6236 |
| 920 | vagina | 17,417 | 8,696 | 432 | 0.9911 | 7.9403 |
| 924 | vagina | 6,366 | 4,305 | 297 | 0.9858 | 7.3824 |
| 928 | vagina | 6,348 | 4,008 | 72 | 0.8522 | 4.2403 |
| 932 | vagina | 3,452 | 2,278 | 23 | 0.6139 | 1.9759 |
| 936 | vagina | 90,700 | 38,090 | 478 | 0.9905 | 8.0971 |
| 948 | vagina | 61,973 | 25,288 | 433 | 0.9888 | 7.8395 |
| 952 | vagina | 5,831 | 3,197 | 104 | 0.6993 | 3.7327 |
| 956 | vagina | 25,515 | 16,637 | 220 | 0.9153 | 5.6878 |
| 960 | vagina | 29,043 | 17,701 | 309 | 0.9704 | 6.9109 |
| 964 | vagina | 60,548 | 42,314 | 23 | 0.1286 | 0.5821 |
| 968 | vagina | 38,994 | 27,776 | 13 | 0.0648 | 0.3153 |
| 972 | vagina | 21,625 | 13,264 | 357 | 0.9893 | 7.725 |
| 976 | vagina | 27,449 | 18,612 | 276 | 0.9682 | 6.9196 |
| 980 | vagina | 4,076 | 2,593 | 82 | 0.7769 | 3.5689 |
| 984 | vagina | 22,769 | 14,766 | 218 | 0.931 | 5.8328 |
| 988 | vagina | 6,712 | 3,563 | 156 | 0.8933 | 5.1188 |
| 992 | vagina | 30,200 | 19,284 | 250 | 0.8988 | 6.0442 |
| 996 | vagina | 49,410 | 29,456 | 415 | 0.9904 | 7.887 |
| 1000 | vagina | 21,306 | 14,671 | 88 | 0.8591 | 3.9628 |
| 1004 | vagina | 14,976 | 9,104 | 262 | 0.9587 | 6.7424 |
| 1008 | vagina | 24,177 | 15,187 | 232 | 0.9712 | 6.5752 |
| 1012 | vagina | 24,643 | 12,774 | 365 | 0.9896 | 7.614 |
| 1016 | vagina | 29,936 | 18,143 | 345 | 0.9888 | 7.6086 |
| 1020 | vagina | 18,636 | 12,224 | 208 | 0.9564 | 6.3466 |
| 1024 | vagina | 45,896 | 21,427 | 486 | 0.9927 | 8.1951 |
| 1028 | vagina | 20,175 | 13,308 | 199 | 0.952 | 5.6872 |
| 1032 | vagina | 35,791 | 26,277 | 125 | 0.7918 | 4.1747 |
| 1036 | vagina | 3,344 | 1,611 | 78 | 0.873 | 4.3176 |
| 1040 | vagina | 8,147 | 4,587 | 321 | 0.9835 | 7.2962 |
| 1044 | vagina | 36,814 | 24,694 | 224 | 0.9512 | 6.1243 |
| 1048 | vagina | 36,948 | 19,044 | 477 | 0.9916 | 8.1727 |
| 1052 | vagina | 35,909 | 23,139 | 260 | 0.9449 | 6.2479 |
| 1056 | vagina | 21,256 | 10,407 | 404 | 0.9889 | 7.7368 |
| 1060 | vagina | 28,540 | 16,500 | 390 | 0.989 | 7.6434 |
| 1064 | vagina | 21,729 | 12,193 | 421 | 0.9925 | 8.012 |
| 1068 | vagina | 3,935 | 2,544 | 128 | 0.8666 | 5.0239 |
| 1072 | vagina | 37,714 | 22,636 | 354 | 0.9874 | 7.5598 |
| 1076 | vagina | 6,236 | 3,744 | 261 | 0.9683 | 6.8294 |
| 1080 | vagina | 10,999 | 5,757 | 422 | 0.992 | 8.0011 |
| 1084 | vagina | 15,524 | 8,128 | 387 | 0.9837 | 7.4871 |
| 1088 | vagina | 2,149 | 1,357 | 54 | 0.7635 | 3.0855 |
